# Chiral Diol-Based Organocatalysts in Enantioselective Reactions

**DOI:** 10.3390/molecules23092317

**Published:** 2018-09-11

**Authors:** Truong N. Nguyen, Po-An Chen, Krit Setthakarn, Jeremy A. May

**Affiliations:** 1 Department of Chemistry, University of Houston, 3585 Cullen Boulevard, Fleming Building Room 112, Houston, TX 77204-5003, USA; tnnguyen61@uh.edu (T.N.N.); pchen25@central.uh.edu (P.-A.C.); 2 Department of Chemistry, Faculty of Science, Silpakorn University, Nakhon Pathom 73000, Thailand; krit_set@silpakorn.edu

**Keywords:** asymmetric catalysis, organocatalysts, organoboronates, allylation, conjugate addition, BINOL, TADDOL, diol catalyst

## Abstract

Organocatalysis has emerged as a powerful synthetic tool in organic chemistry in the last few decades. Among various classes of organocatalysis, chiral diol-based scaffolds, such as BINOLs, VANOLs, and tartaric acid derivatives, have been widely used to induce enantioselectivity due to the ability of the hydroxyls to coordinate with the Lewis acidic sites of reagents or substrates and create a chiral environment for the transformation. In this review, we will discuss the applications of these diol-based catalysts in different types of reactions, including the scopes of reactions and the modes of catalyst activation. In general, the axially chiral aryl diol BINOL and VANOL derivatives serve as the most competent catalyst for most examples, but examples of exclusive success using other scaffolds, herein, suggests that they should not be overlooked. Lastly, the examples, to date, are mainly from tartrate and biaryl diol catalysts, suggesting that innovation may be available from new diol scaffolds.

## 1. Introduction

**Scheme 1:**
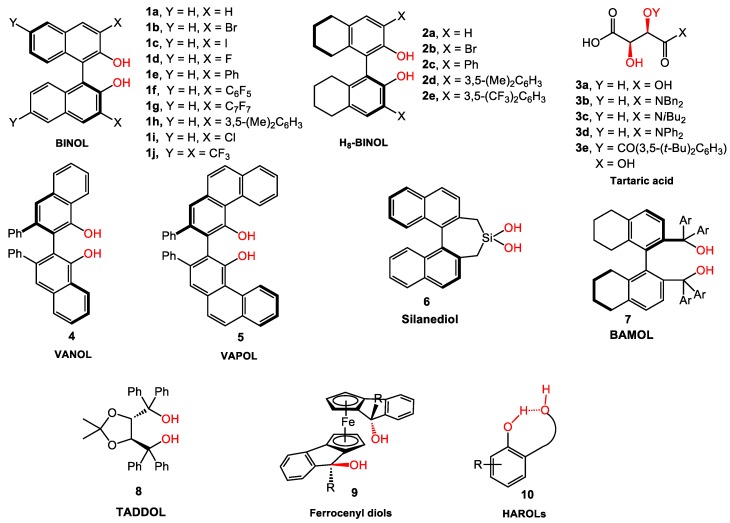
Common chiral diol-based organocatalysts.

Although the use of small organic molecules to catalyze organic transformations, especially in an enantioselective manner, has been sporadically reported in the literature since the 1970s [1], it was not until 2000 that the term “organocatalysis” was popularized, and the field grew exponentially. Organocatalysis provides several advantages relative to transition-metal catalysis and enzyme-based catalysis that include low toxicity, ready availability of chiral pool catalyst building blocks, insensitivity to moisture and air, and selectivity with well-defined organization in transition states. Over the years, several classes of organocatalysts have been developed to enable enantioselective reactions through either covalent catalysis (iminium, enamine, Lewis base, and SOMO catalysis) or non-covalent catalysis (ion-pairing, hydrogen-bonding, and Brønsted acid/base catalysis) [2,3,4,5,6]. Among the catalysts that activate the substrate through transient interactions, diol-based catalysts, such as derivatives of BINOL and TADDOL (Scheme 1) have widely served as versatile chiral tools to catalyze many different types of reactions. Although they have been significantly used as chiral ligands in transition-metal catalysis and Lewis acid complexes, the unfettered hydroxyls of these catalysts can, themselves, facilitate certain reactions. This review exclusively covers applications of the catalysts where reactivity is induced and the stereoselectivity is controlled through substrate or reagent activation by the hydroxyls of the diols. The principle discussion focuses on asymmetric transformations that involve the interaction of the hydroxyls with organoboron reagents, as well as reactions driven by activation through non-covalent interactions. The presentation is organized based on the diol scaffold: BINOL, tartaric acid derivatives (those catalyzed by carboxylic acid group are not covered), VANOL/VAPOL, silanediol, TADDOL, BAMOL, ferrocenyl diol, and HAROL derivatives (Scheme 1).

## 2. BINOL Derivatives

### 2.1. Allylboration

#### 2.1.1. Allyboration/Crotylboration of Ketones

**Scheme 2:**
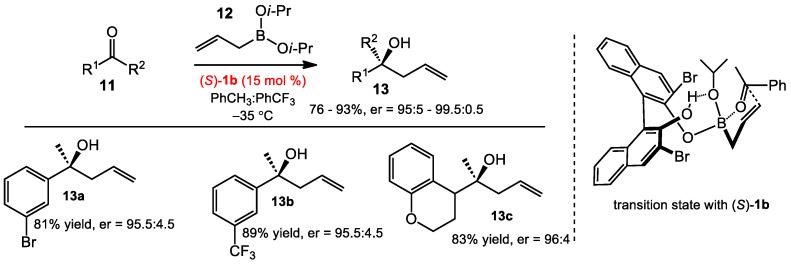
Asymmetric allylboration of ketones and proposed transition state.

One of the most common synthetic transformations in organoboron chemistry is allylboration. Despite the existing methods for asymmetric allylation of ketones such as allyl stannanes catalyzed by Ti complexes [7] or Ag catalysts [8], and allyl boronic esters via Cu(II) catalysis [9], the enantioselective organocatalytic allylboration of ketones was unknown until the work of Schaus in 2006 with chiral BINOL derivatives [10] (Scheme 2). Based on the postulate that diol catalysts could serve as exchangeable chiral ligands with organoboronates, the authors investigated that allyldiisopropoxylborane **12** underwent allylboration to ketones **11** in the presence of a catalytic amount of BINOL **1b** (15 mol%). Optimal results were obtained when running the reaction in a mixture (1:3) of PhCH_3_ and PhCF_3_ at −35 °C, affording tertiary homoallylic alcohols up to 93% yield and 99% ee. The reaction conditions were able to facilitate allylboration of both electron-rich and electron-deficient aromatic ketones (see **13a**–**c**).

**Scheme 3:**
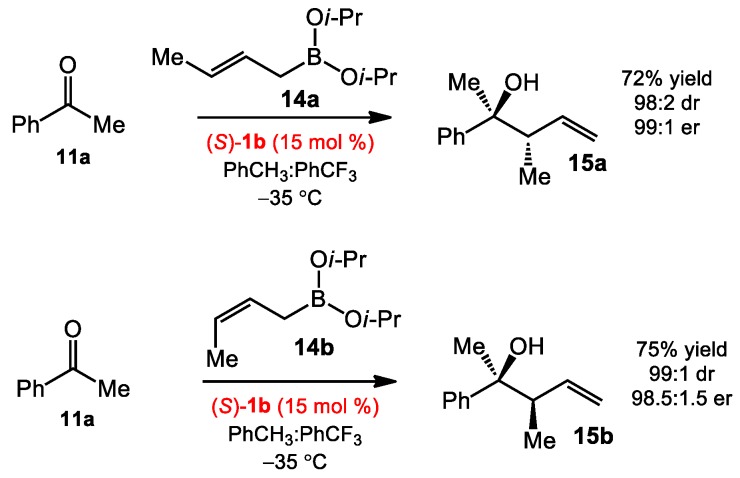
Asymmetric crotylboration of acetophenone.

The catalyst **1b** also allowed crotylboration of acetophenone in good yields and high selectivities (Scheme 3). (*E*)-crotylboronate **14a** afforded the *anti*-product **15a**, while (*Z*)-crotylboronate **14b** gave the *syn*-product **15b**. These results were consistent with a Zimmerman–Traxler model. On the basis of mechanistic experiments, the authors proposed that a catalyst-associated boronate complex was formed though rapid exchange of one isopropoxyl group, followed by the coordination of the Lewis acidic boron atom to the ketone. The enantioselectivity was proposed to be controlled by the hydrogen-bonding interaction between the alkoxy ligand and the “free” hydroxyl group of the catalyst, leading to *si* facial attack via a chair-like transition state (Scheme 2). These preliminary mechanistic understandings of the activation modes of diol catalysts opened the door for the asymmetric reaction development using organoboronates later on.

#### 2.1.2. Allyboration/Crotylboration of Preformed Acyl Imines

**Scheme 4:**
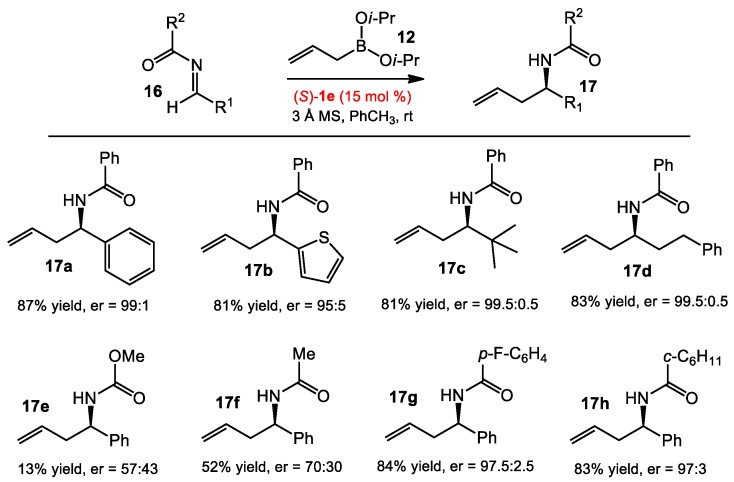
Asymmetric allylation of acyl imines.

With the success of using BINOL to catalyze asymmetric allyboration of ketones, the Schaus group continued to expand this catalytic system to acyl aldimines (Scheme 4) [11]. The authors found that allylborane **12** was viable for the allylboration of acyl aldimines **16** with high enantioselectivity, by modifying the conditions for ketones. The use of a 3,3′-Ph_2_-BINOL catalyst afforded the highest enantioselectivity and reactivity. Non-polar solvents, like toluene, were found to improve the results. However, the key modification was the addition of 3 A molecular sieves, which presumably prevented the hydrolysis of acyl imines by trace amounts of water. Regarding the reaction scope, most benzoyl imines—including heteroaromaric imines and aliphatic imines—were excellent substrates for the reaction (see **17a**–**d**). However, the nature of the acyl group significantly affected the results. The reaction of methyl carbamate afforded the product **17e** in only 13% yield and 57:43 er, presumably due to the decomposition of carbamoyl imines via alcoholysis. Replacing the benzoyl imine with acetyl imine resulted in low selectivity and reactivity in the product **17f** (52% yield, 70:30 er), while a larger acyl group gave similar results to those of benzoyl imines (see **17h**). Electron-deficient acyl imines were also found to be good substrates for allylboration (see **17g**).

**Scheme 5:**
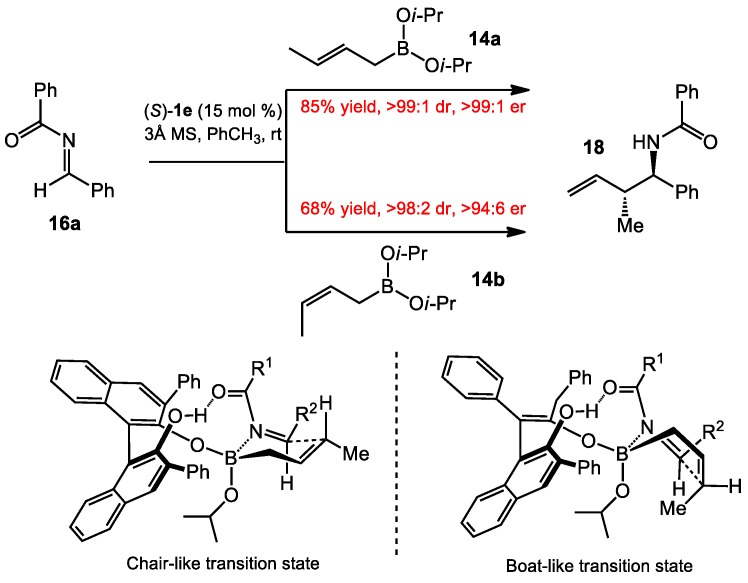
Asymmetric crotylboration of benzoyl imines and proposed transition state.

The crotylboration of acyl imines was also investigated with the optimized conditions. The authors found a surprising result. Both (*E*)- and (*Z*)-crotylboronates **14** provided the *anti*-product **18** with high diastereoselectivity and enantioselectivity (Scheme 5). The formation of the *anti*-product from the (*E*)-crotylboronate **14a**, could be rationalized via a chair-like transition state using the (*Z*)-hydrazone conformer, which had been proposed previously [12,13]. We have recreated the Schaus’s proposed transition state models in Scheme 5, though we note there is an odd relationship with the “front” hydroxyl hydrogen bonding to the amide carbonyl in the “back” of the structure. With the (*Z*)-crotylboronate **14b**, a boat configuration in the transition state was presumably preferred, due to the diaxial interaction of the methyl group and the acyl group in a chair transition state, which caused transition to a boat-like transition state, to provide the same product diastereomer, and the newly formed allylic stereocenter would be inverted relative to what would be predicted with the Zimmerman–Traxler model. An alternative hypothesis to explain that the (*Z*)-crotyl boronate also gives the *anti*-diastereomer is that the same diaxial interaction mentioned in the previous sentence could cause an extremely slow reaction to occur, allowing time for *E*/*Z* isomerization of the hydrazone, and the (*E*)-hydrazone reacts with the (*Z*)-crotylboronate. Putting the (*E*)-hydrazone in the Zimmerman–Traxler model would exchange the H and R^2^ groups in the chair-like transition state in Scheme 5 and, thus, avoid diaxial interactions. The result of this change would be that the newly formed benzylic amine stereocenter would be inverted in the product. Assuming that the transition state organization by the BINOL catalyst is consistent for both (*E*)- and (*Z*)-crotyl nucleophiles, the fact that the major enantiomer is the same for both isomers strongly suggests that the boat-like transition state is correct, since the sense of enantioenduction for the amine stereocenter is the same in both cases. The well-ordered coordination of the diol–boronate complex to acyl imines is facilitated by hydrogen-bonding interactions, allowing the facial selective addition of crotylboronates. Hydrogen-bonding interactions, including hydrogen-bonding networks between proximal hydroxyls, have been shown to have substantial effects in aprotic solvents that include the lowering of pK_a_’s and the ability to bind anionic and Lewis basic atoms [14,15,16].

#### 2.1.3. One-Pot Allyboration/Crotylboration of Acyl Imines Derived from Free Aldehydes

**Scheme 6:**
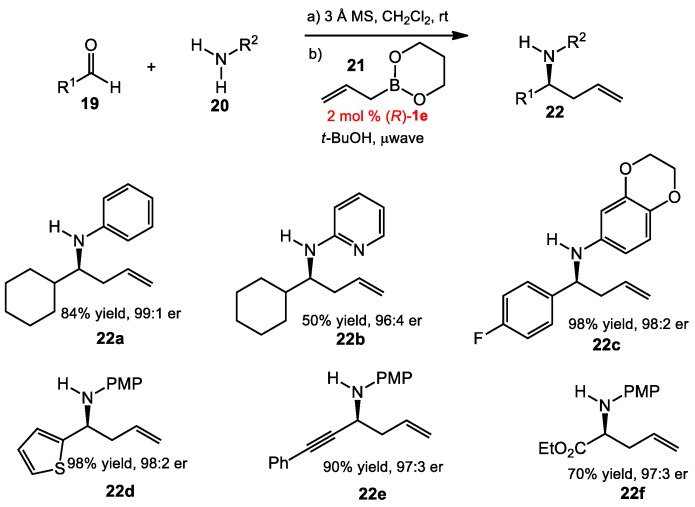
Asymmetric Petasis allylation.

The multicomponent reaction between α-hydroxyl aldehydes, organoboronic acids, and amines, is known as the Petasis reaction [17]. A subclass of this reaction is the uses of allylboronates as nucleophiles. Although progress towards the enantioselective allylation of in situ-generated imines has been made [18,19], an asymmetric allylboration reaction of any desired imine remains challenging. Recently, Schaus and co-workers reported a general strategy for enantioselective Petasis-like allylation reactions of various aldehydes and amines using chiral diol catalysis [20]. During the course of optimization, a one-pot reaction was developed (Scheme 6). Aldehydes **19** and amines **20** were mixed in the presence of 3 A molecular sieves to form imines, followed by the addition of catalyst **1e**, *t*-BuOH, and cyclic allyboronate **21**, with the assistance of microwave irradiation at 50 °C to afford homoallylic amines **22** in good yields and at high enantioselectivities. Compared to the previous allylboration reaction of acyl imines, this procedure provided a much larger scope with a wide structural and electronic range of imines. In general, electron-deficient substrates gave products with higher yields than those from electron-rich substrates. Aliphatic and heteroaromatic aldehydes, as well as ethyl glyoxylate, were also good substrates. Different amines can be employed with similar outcomes.

**Scheme 7:**
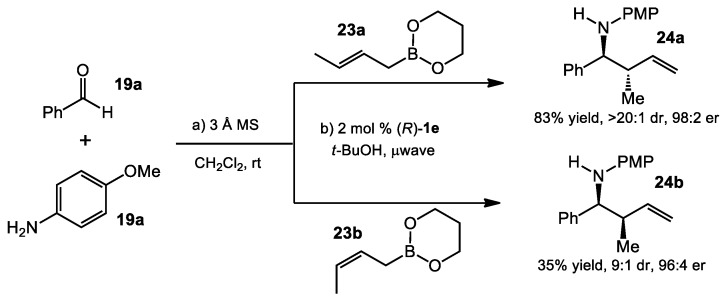
Asymmetric Petasis crotylation.

The optimized conditions were applied to crotylboration reactions (Scheme 7). Although the stereochemical outcome of this case could be complicated due to the involvement of many factors (e.g., chair-like or boat-like transition state, *E*/*Z*-imine isomerization prior to crotylboration), the reactions were consistent with the Zimmerman–Traxler transition state model. (*E*)-crotylboronate **23a** provided the *anti*-adduct **24a** with high diastereo- and enantioselectivity (>20:1 dr and 98:2 er), while (*Z*)-crotylboronate **23b** gave the opposite diastereomer **24b** in lower yield and dr (35% yield, 9:1 dr).

#### 2.1.4. Traceless Petasis Borono–Mannich Allylation/Crotylation

**Scheme 8:**
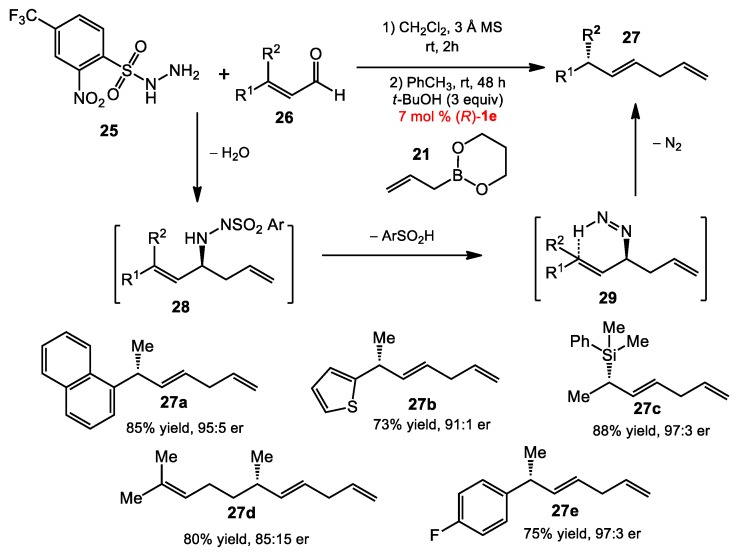
Reductive allylation of enals.

Chirality transfers via allylic diazene rearrangements have been employed in synthetic method development [21,22]. The existing methods usually involved the fragmentation of sulfonyl hydrazine precursors to generate in situ diazenes, followed by a retro-ene reaction with the loss of nitrogen gas. Despite significant advances, these reactions relied on optically active starting materials to transfer chirality to the final products [23,24]. Recently, the Thomson and Schaus groups reported a method that overcame this limitation [25]. Chiral allylic diazenes could be directly accessed through the diol-catalyzed asymmetric allyboration of allylic hydrazones. Subsequent rearrangement resulted in a 1,3-transposition of chirality to the allylic carbon center (Scheme 8), affording enantioenriched 1,4-diene products. The reaction between the electron-deficient hydrazide **23**, enal **24**, and allylboronate **21** under the modified conditions for Petasis allylation (7 mol% **1e**, 3 equiv of *t*-BuOH in toluene at room temperature) provided the optimal results. The conditions were suitable with a variety of enals, even a silylmethyl enal (see **27c**).

**Scheme 9:**
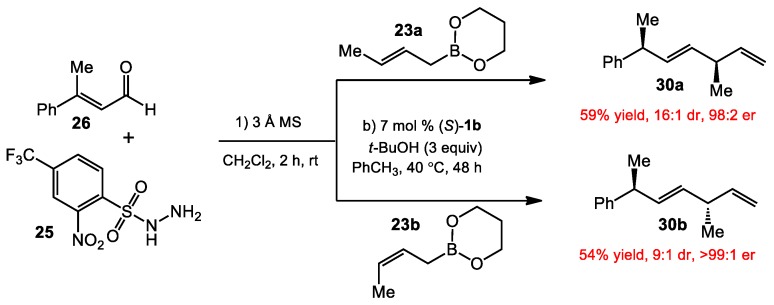
Diastereoselective reductive crotylations.

The crotylboronates **23** were subjected to the conditions to reveal the reaction’s diastereoselectivity. As anticipated, the diene products **30**, bearing two methyl-substituted stereocenters with a *syn* or *anti* stereochemical relationship, were produced in good yields and with high levels of stereocontrol (Scheme 9). Notably, the stereoselective outcome was consistent with those of the asymmetric Petasis crotylborations [20]. The poor diastereoselectivity for **30b** generated from (*Z*)-crotylboronate could be rationalized by an unfavorable 1,3-diaxial interaction between the methyl group of the boronate and the sulfonyl group in the transition state [11].

#### 2.1.5. Methallylation

**Scheme 10:**
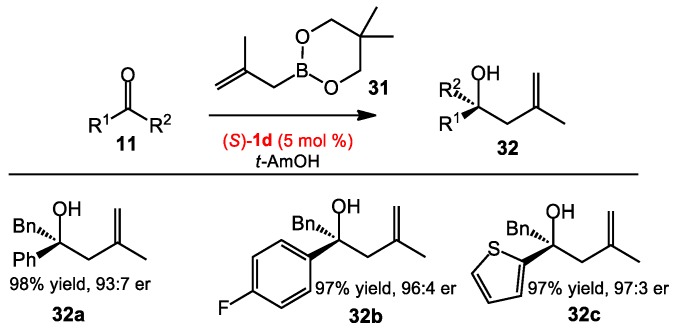
Asymmetric methallylation of ketones.

Although allylation and methallylation reactions of ketones are seemingly similar processes, the conditions developed for the allylation of ketones are often not directly applicable to methallylation processes, due to differential demands of the vinyl substitution [26]. Given the synthetic significance of the asymmetric methallylation of ketones, there was a need to develop general conditions for this transformation. In 2013, Zhang and co-workers reported an efficient approach for the enantioselective methallylboration of ketones catalyzed by a new BINOL catalyst [27]. The authors found that the reaction was sluggish when the allylboration conditions reported by Schaus were applied. During modification of the catalyst, 3,3′-F_2_-BINOL was observed to give the best results, while 3,3′-(CF_3_)_2_-BINOL surprisingly gave no enantioselectivity. The new optimized conditions were effective for a variety of ketones (Scheme 10).

#### 2.1.6. Allylboration of Ketones with γ-Disubstituted Allylboronic Acids

**Scheme 11:**
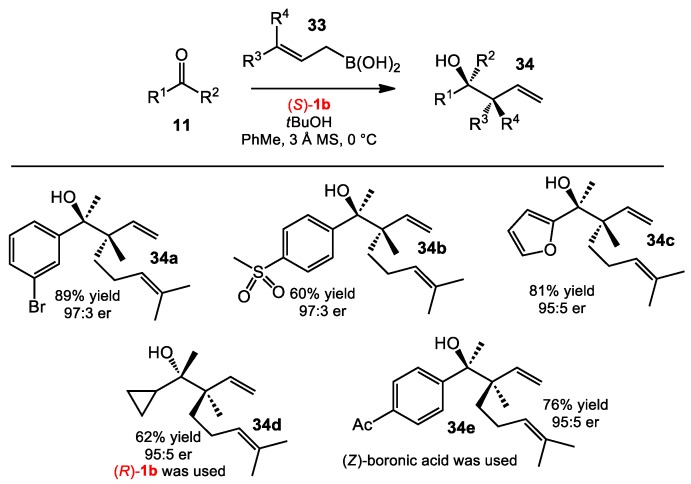
Allylboration of ketones with geranylboronic acid.

The synthesis of two adjacent tetrasubstituted stereocenters is extremely challenging, especially in an enantioselective fashion. By using γ-disubstituted allylboronic acids as nucleophiles for the allylboration of ketones, such synthetic motifs can be obtained when using BINOL catalysts [28]. The Szabó group showed that γ-disubstituted boronic acids **33** reacted with ketones **11** when applying the conditions developed by the Schaus group. With an appropriate choice of BINOL catalysts and stereoisometric substrates, all four possible enantiomers of homoallylic alcohols **34** were synthesized. The conditions were also suitable for a large range of ketones, including aliphatic and heteroaromatic ketones (Scheme 11).

It is notable that, to date, the catalysts derived from BINOL have been the most effective for allylation and crotylation reactions. This has been true whether the electrophile is an aldehyde, ketone, imine, or hydrazone, which suggests a privileged status for BINOL catalysis in enantioselective reactions allylic boronates.

### 2.2. Propargylation

**Scheme 12:**
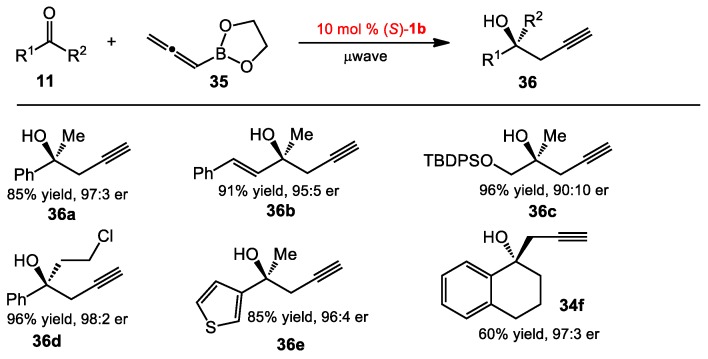
Asymmetric propargylation of ketones.

The 3,3′-Br_2_-BINOL **1b** was also able to catalyze the asymmetric propargylation of ketones when using allenylboronate **35** as the nucleophile, under microwave irradiation and in the absence of solvent, to provide homopropargylic alcohols **36**, versatile building blocks in organic synthesis (Scheme 12) [29]. The products were obtained in a range of good to excellent yields and enantioselectivities (60–98% yield, 3:1–99:1 er). Aromatic ketones were generally good substrates, affording products with higher enantioselectivities compared to those from aliphatic ketones. Heteroaromatic and cyclic ketones were also effective in the reaction conditions (see **36e**–**f**).

**Scheme 13:**
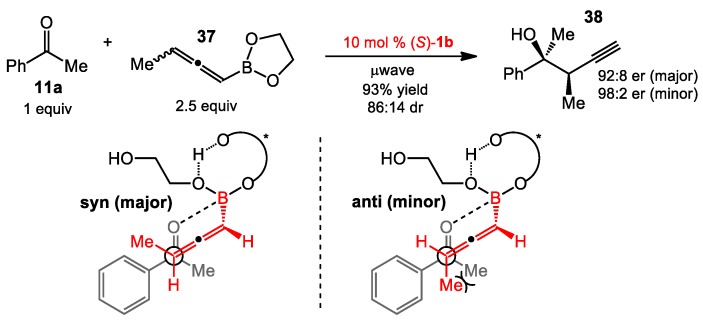
Diastereoselective propargylation of ketones.

Racemic methylallenylboronate **37** was also used in the reaction to study the diastereoselectivity. The *syn*-product **38** was obtained as the major diastereomer (84:16 dr). The result could be explained via the model illustrated in Scheme 13. The *syn*-product arose from a preference to avoid a gauche interaction between the two methyl groups in acetophenone and allenylboronate. Both diastereomers were obtained with high enantioselectivities (92:8 er). Like the allylations of ketones, aldehydes, and imines discussed above, the only catalyst scaffold that has been reported, to date, for enantioselective propargylation using allenyl boronates, has been a derivatized axially chiral aromatic diol. Both VANOL and BINOL derivatives were catalytically competent, with the BINOL derivatives giving higher degrees of stereoselectivity. Results for other organic diols discussed in this review were not presented in the propargylation report, though it is clear that the authors were aware of other catalysts, given their work in additions to acyl quinoliniums (Section 3.1 of this review).

### 2.3. Addition of Organoboronates to Acyl Imines

**Scheme 14:**
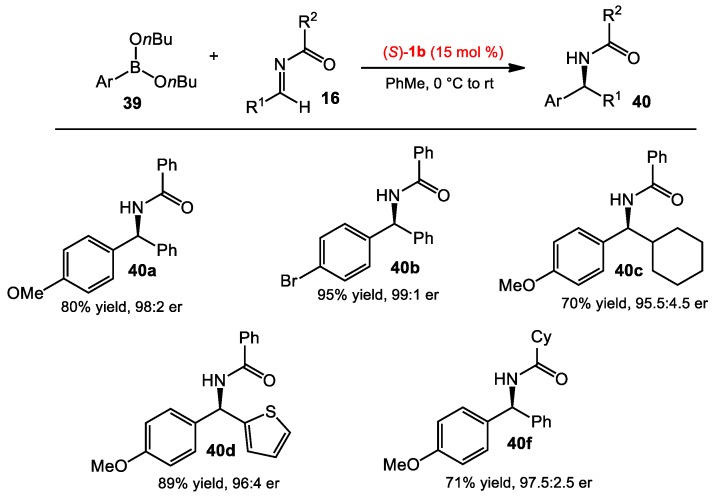
Asymmetric arylboration of acyl imines.

**Scheme 15:**
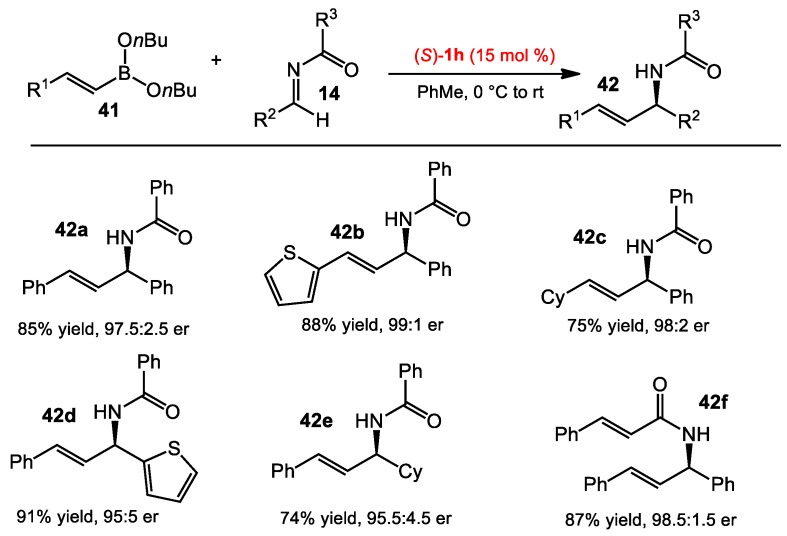
Asymmetric vinylboration of acyl imines.

**Scheme 16:**
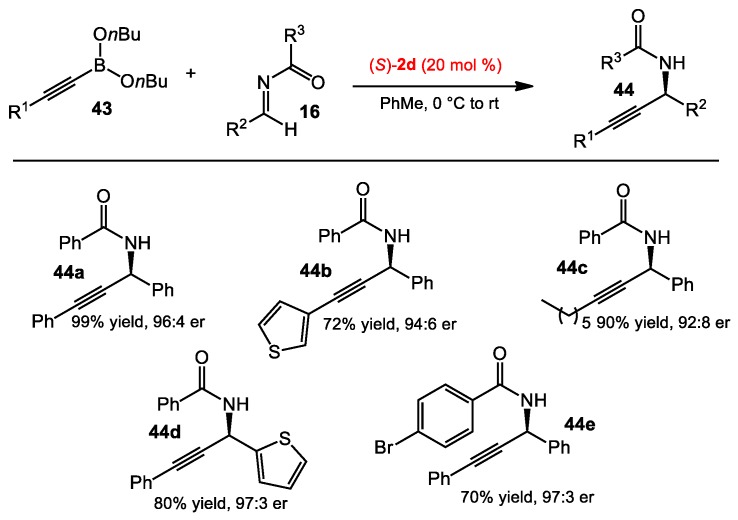
Asymmetric alkynylboration of acyl imines.

Beyond the asymmetric allylboration of acyl imines, Schaus and co-workers also sought suitable conditions to vinyl, alkynyl, and aryl boronate nucleophiles. After the optimization process, *n*-butyl boronate was used due to its hydrolytic stability. A catalyst screening identified a suitable BINOL catalyst for the addition of each type of nucleophile, including aryl, alkenyl, and alkynylboronates to acyl imines [30]. The BINOL **1b** was found to be optimal with arylboronates (Scheme 14), whereas catalyst **1h** and **2d** were more suited for alkenylboronates and alkynylboronates, respectively (Scheme 15 and Scheme 16). The scope of the reaction was examined for each class of boronate nucleophiles. The optimized conditions were effective for arylboronates, aryl and aliphatic amines, and different acyl substituents, affording the corresponding products in good yields (>70%) and enantioselectivities (>95:5 er). Vinylboronates **41** also provided the allylic amine products **42** in similar reactivities and selectivities to those of arylboronates. Propargyl amines **44** could be obtained in good yields and selectivities from substituted alkynylboronates **43**.

**Scheme 17:**
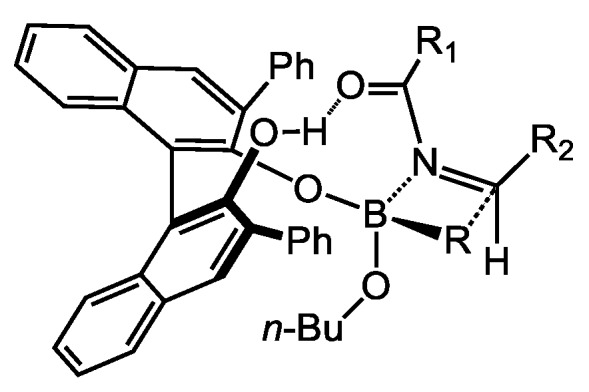
Proposed transition state for asymmetric boronate addition.

Although computational studies suggested the formation of a cyclic boronate complex [31,32], the authors believed that an acyclic boronate complex was formed under the reaction conditions based on their mechanistic experimental results. A stereochemical model was made to rationalize the observed stereoinduction (Scheme 17). The boronate complex coordinates to the (*Z*)-conformer of the acyl imine. The hydrogen-bonding interaction between an uncoordinated hydroxyl of the BINOL and the acyl group was proposed to orient the nucleophilic attack to the acyl imine and, thus, control the facial selectivity.

### 2.4. Addition of Boronates to o-Quinone Methides

**Scheme 18:**
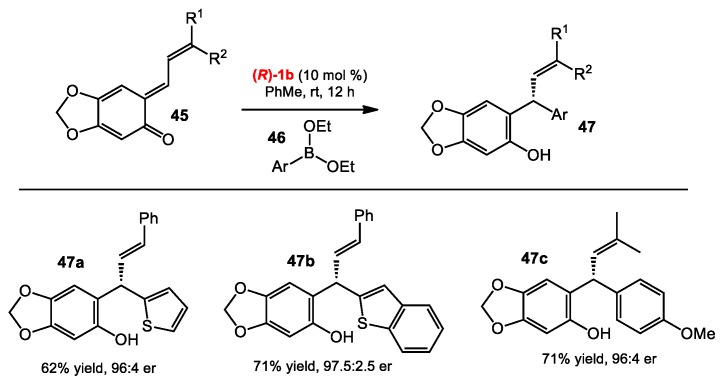
Enantioselective addition of boronates to vinyl *o*-QMs.

**Scheme 19:**
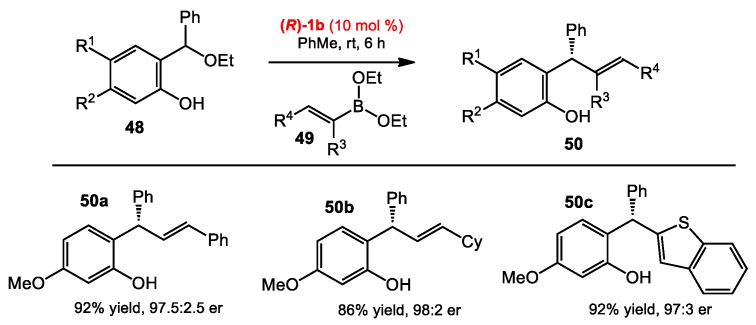
Enantioselective addition of boronates to hydroxybenzyl ethyl ethers.

*o*-Quinone methides (*o*-QMs) have been employed not only as the building blocks for hetero-Diels–Alder reactions [33], but also as electrophiles of 1,4-conjugate additions at the exocyclic carbon [34]. Inspired by the asymmetric nucleophilic addition of organoboronates catalyzed by BINOL catalysts, Schaus and co-workers developed catalytic enantioselective reactions between boronates and *o*-QMs catalyzed by chiral diols [35]. The reaction of *o*-QMs **45** and arylboronates **46** gave the optimal outcome when using catalyst **1b** in toluene at room temperature. The conditions were successfully used with a wide range of arylboronates, even with heteroarylboronates, affording products **47** in similar yields and enantioselectivities (Scheme 18). Alkenylboronates were also found to be effective. Next, the authors expanded the scope of *o*-QMs by generating in situ *o*-QMs from easily accessible precursors. Hydroxyl-substituted benzyl alcohols have been known to generate *o*-QMs under acidic conditions. Indeed, the reaction between hydroxylbenzyl alcohols **4** and alkenylboronates **49** in the presence of the catalyst **1b** afforded products **50**, presumably because the boronates **49** are acidic enough to promote the formation of *o*-QMs from **48** (Scheme 19). Notably, the ortho hydroxyl group of the phenol was found to be crucial, as no product was observed in its absence. Under the optimized conditions, the products **50** were obtained in excellent yields, and with high enantioselectivities from either electron-rich or electron-deficient substrates.

### 2.5. Multicomponent Quinone Methide Condensation

**Scheme 20:**
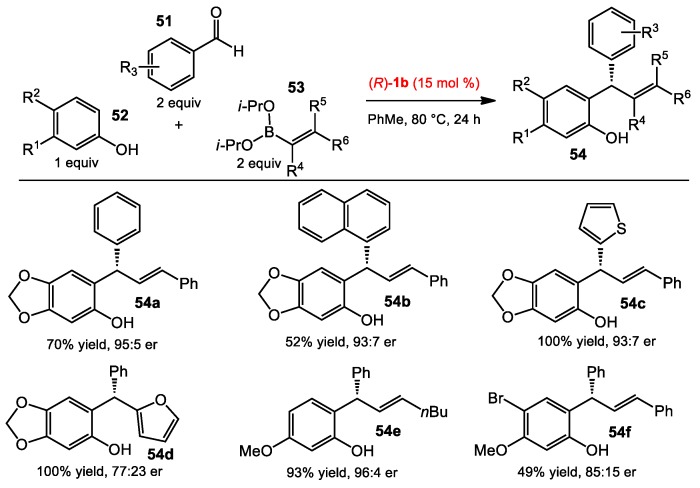
Multicomponent boronate condensation reactions.

**Scheme 21:**
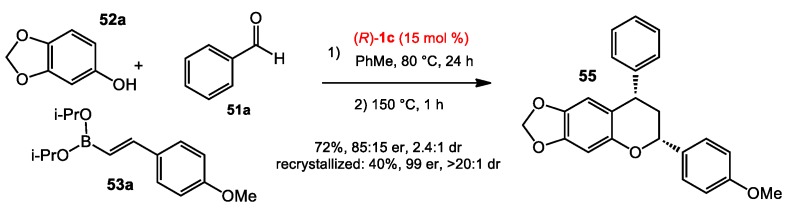
Chroman synthesis.

With the success of the capture of in situ-generated *o*-QMs by nucleophilic additions of boronates, the Schaus group also conceived an enantioselective multicomponent reaction from phenols, aldehydes, and boronates to afford chiral scaffolds found in natural products and drugs [36]. *o*-QMs would be formed via the condensation of phenols and aldehydes mediated by boronates, followed by the asymmetric nucleophilic addition of the organoboronates to the methide carbon catalyzed by the chiral diol. The reaction investigation started with an electron-rich phenol **52**, an aldehyde **53**, and a styrenylboronate **54**. After screening conditions, the optimal result was achieved when using catalyst **1b** at 80 °C in toluene (Scheme 20). These optimized conditions were suitable to a wide range of aromatic aldehydes. However, the phenol **52** needs to be electron-rich to facilitate the reaction. Both alkenyl and arylboronates provided the products in good to excellent yields and enantioselectivities, though some heteroarylboronates performed less well (see **54d**). Interestingly, a chroman product was obtained when using 4-methoxylstyrenylboronate as the nucleophile (Scheme 21). This result led the authors to optimize the reaction conditions towards the formation of the chroman product. By changing to catalyst **1c** and heating the reaction at 150 °C for 1 h after the standard conditions, the chroman adducts were obtained in good yields and moderate selectivities. The levels of diastereo- and enantiopurity of the products could be improved after recrystallization.

**Scheme 22:**
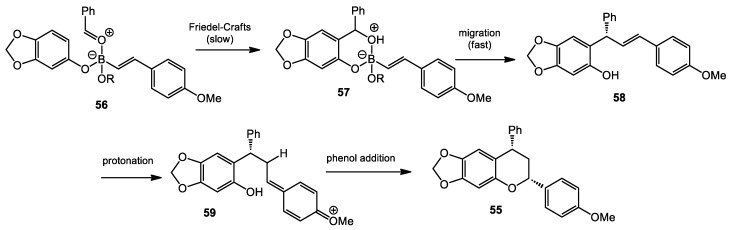
Proposed mechanism for chroman formation.

The authors also proposed a reaction mechanism based on their observations (Scheme 22). The Lewis acid/Lewis base complex **56** was formed by the condensation of the phenol, aldehyde, and styrenyl boronate, followed by Friedel–Crafts alkylation mediated by the Lewis acidic boron atom to afford **57**. This step was proposed to be the rate determining step, as no products were formed at a lower temperature. The complex **58** then associated with the catalyst in an unclear organization, allowing the facially selective migration of the alkenyl group to afford enantioenriched **59**. Whether this would be a direct displacement akin to a Matteson reaction, or a stepwise process through a benzylic cation/orthoquinone methide, remains unproven, but is most likely the latter, due to unlikely orbital overlap for a Matteson-like migration. Next, the protonation of the olefin initiated the cyclization reaction, followed by nucleophilic addition/rearomatization to provide the chroman **55**.

### 2.6. Allene Formation via Allylboration and Alkynylboration

**Scheme 23:**
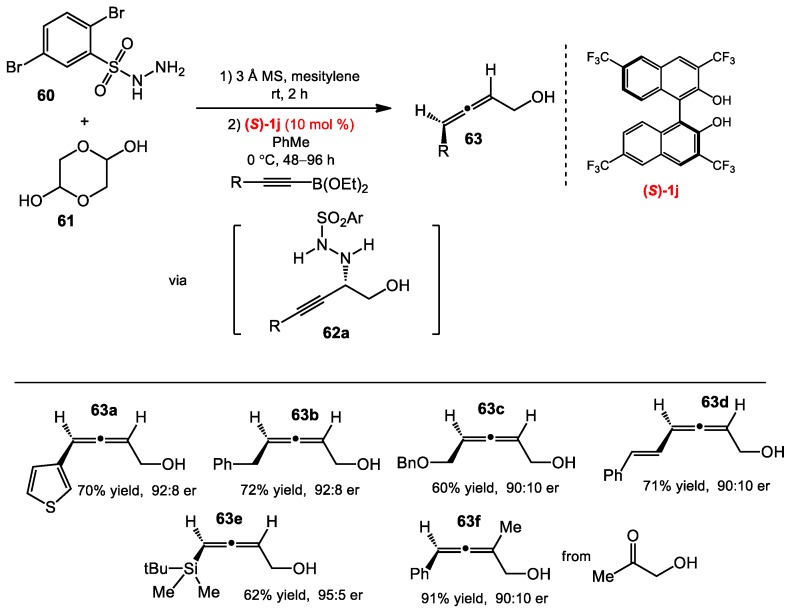
Allene synthesis via alkynyl boronates.

**Scheme 24:**
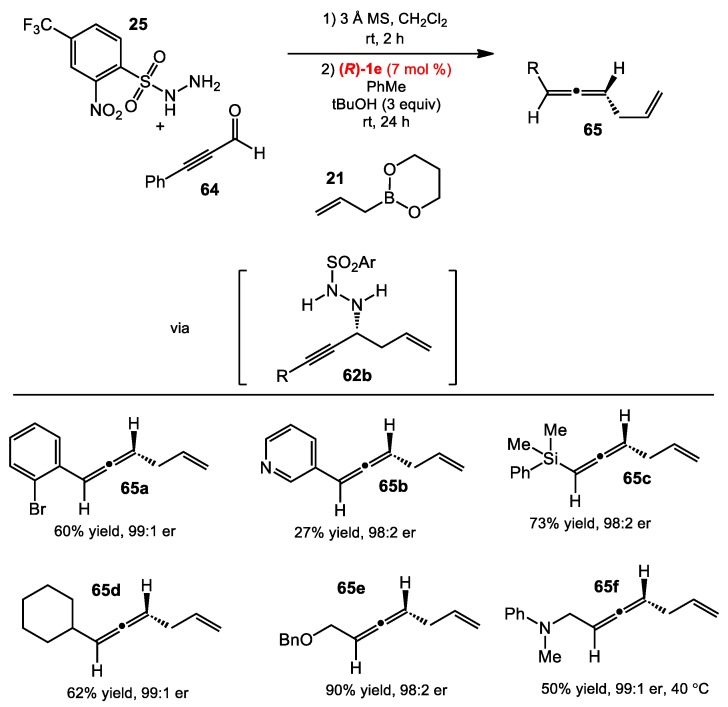
Allene synthesis from alkynyl aldehydes.

In 2012, Thomson and co-workers [37,38] reported a strategy to synthesize racemic allenes that they called a traceless Petasis reaction. The addition of alkynyltrifluoroborates to sulfonylhydrazones generated propargylic hydrazide intermediates. The hydrazides spontaneously eliminated sulfinic acid to generate propargylic diazenes that decomposed by a retro-ene reaction to form the allene products. Based on the success of this reaction, Schaus and Thomson envisioned that an asymmetric version of the traceless Petasis reaction could be developed by devising a strategy to access chiral propargylic hydrazides, since the chirality of the propargylic center would be transferred in the retro-ene reaction [39]. They developed two different approaches towards optically active propargylic hydrazides: asymmetric alkynylboration of transient α-hydroxyl hydrazones (Scheme 23) and asymmetric allylboration of transient propargylic hydrazones (Scheme 24). In the former case, the hydroxyl group was found to be crucial for an effective reaction for its coordination to alkynylboronates, while it was unnecessary in the latter strategy. During the optimization for the reaction of alkynylboronates and glycolaldehyde, 2,5-dibromophenylsulfonylhydrazide **60** and 3,3′,6,6′-(CF_3_)_4_-BINOL were found to be optimal for the reaction (Scheme 23). The use of a toluene/mesitylene mixture solvent also improved the reaction selectivity. A wide range of arylalkynylboronates was effective under the optimized conditions, affording *α*-allenols **63** in good yields and high enantioselectivities, even with a trialkylsilylalkynyl boronate. Glycolaldehyde could be replaced by α-hydroxylacetone to provide trisubstituted allene **63f** in 91% yield and 90:10 er.

Shaus and Thomson also developed conditions for the allylboration of propargylic hydrazides. The optimal conditions were similar to those for the traceless Petasis allylboration of imines [25,40], which used the sulfonyl hydrazide **25** and the 3,3′-Ph_2_-BINOL catalyst **1e** (Scheme 24). The conditions were effective for both electron-rich and electron-deficient aldehyde substrates. Heteroaromatic propiolaldehydes were also good substrates, though the pyridine-containing aldehyde provided a low yield (27%), presumably because the Lewis basic nitrogen inhibited the reaction.

### 2.7. Conjugate Addition

One very well-known reaction to establish C–C bonds in organic synthesis is the addition of stabilized anionic carbon nucleophiles to electron-deficient olefin acceptors. Various developments and modifications of this reaction have been explored and developed over the centuries. Many approaches to introduce stereoselective versions of these transformations have been reported, including activation of the acceptors and controlled addition of the nucleophiles. Though the use of asymmetric organometallic complexes is a powerful strategy developed for the latter strategy, the preparation of the catalytic complexes requires involved techniques and air-free procedures. Moreover, transition metal catalysts are incompatible with many important functional groups. Therefore, the use of chiral organocatalysis is an important alternative method to promote asymmetric conjugate additions [41].

#### 2.7.1. Conjugate Addition of Alkynyl Boronates

**Scheme 25:**
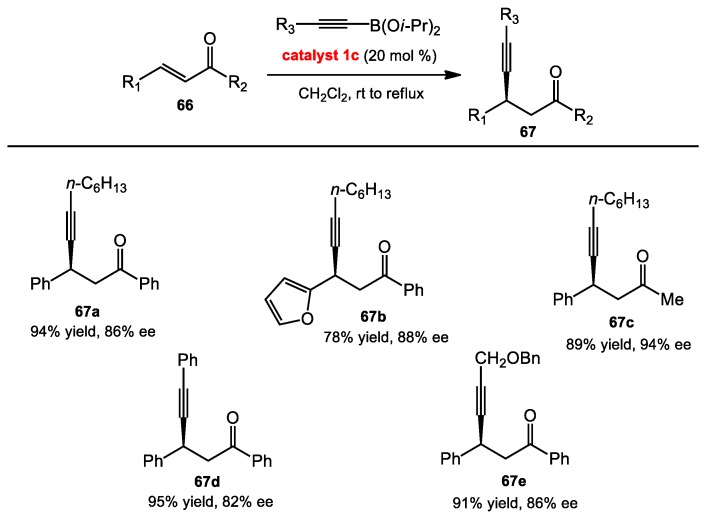
Conjugate addition of alkynyl boronic esters.

The Chong group reported a catalytic conjugate addition using alkynyl boronates (Scheme 25) [42]. They disclosed that the presence of an electron withdrawing group (EWG) substituent at the 3 and 3′ positions of the BINOL positively affected the reaction. Using BINOL with EWGs, the reactions efficiently proceeded and were completed in a shorter time than with no substituents, neutral groups, or EDGs. The most productive results were observed with BINOL catalyst **1c**, which had iodo substituents. Several examples of the enones were shown to react in high yields and enantioselectivities with both alkylalkynyl and arylakynyl boronates as nucleophiles.

#### 2.7.2. Conjugate Addition of Alkenyl Boronates

**Scheme 26:**
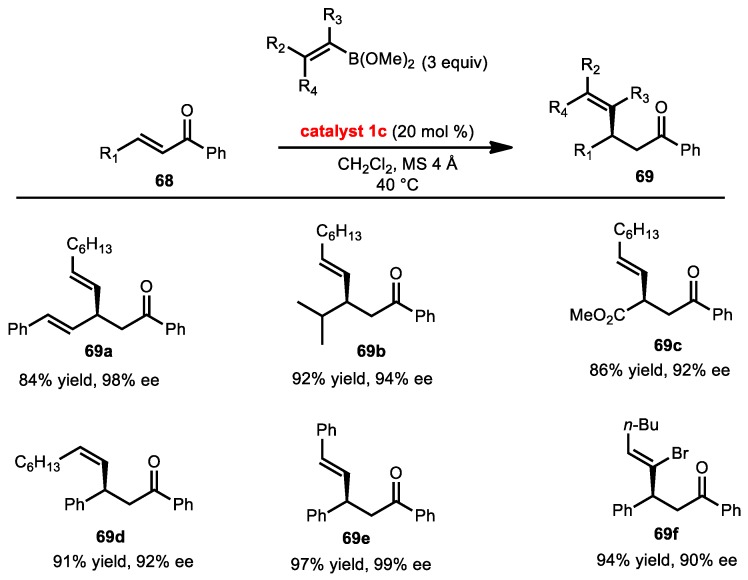
Conjugate addition of alkenyl boronic esters.

The Chong group first reported the enantioselective conjugate addition of alkenyl boronates to chalcones (Scheme 26) [43]. In these studies, they investigated the effect of the 3,3′-substituent of BINOL on enantioinduction. BINOLs with electron-withdrawing groups (I, Br, and CF_3_) provided the best enantiocontrol for the reaction. The ligand bearing iodo substituents was found to be the most effective catalyst, and permitted a lower catalyst-loading compared to other catalysts. Only 1,4-nucleophilic addition to the doubly conjugated enone precursor to **69a** is observed, and it occurs with high enantioinduction. Furthermore, the branched alkyl substituent at the β-position was tolerated to form **69b**. An enone with a carbomethoxy group proceeded favorably to generate a single regioisomer **69c** with high enantiocontrol. Notably, a (*Z*)-boronic ester **69d** exhibited an excellent enantioselectivity with retention of configuration of the alkene.

**Scheme 27:**
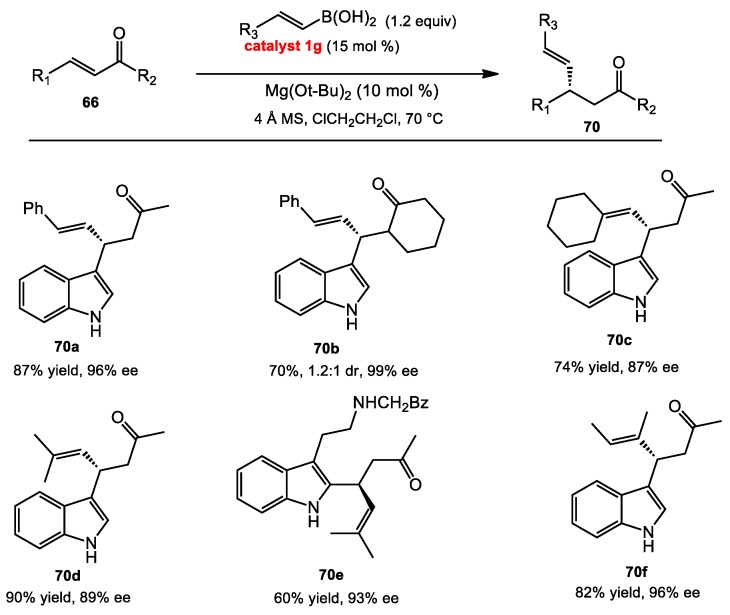
Conjugate addition of alkenyl boronic acids.

Later on, Chong’s work inspired our group to develop the conjugate addition with β-indolo-enone substrates. Enantioselective conjugate addition to β-indolo-enones had been quite rare, since the indolo-enone is unreactive. Chong’s reaction conditions synthesized the conjugate adduct **70f** in poor yield. We sought out a stronger Lewis acidic catalyst to improve the catalytic reactivity of the BINOL catalyst and, thus, increase the reactivity of the enones. Our group found that instead of using iodo substituents on BINOL, a 3,3′-C_6_F_5_-substituted BINOL was found to be a more reactive catalyst for the conjugate addition of alkenyl boronic acids to β-indolo-enones (Scheme 27) [44]. Acyclic and cyclic alkenyl boronic acids provided products in high yield and enantioselectivity. Notably, these reaction conditions proceeded without the need of protecting groups on the indole.

#### 2.7.3. Conjugate Addition of Aryl Boronates

**Scheme 28:**
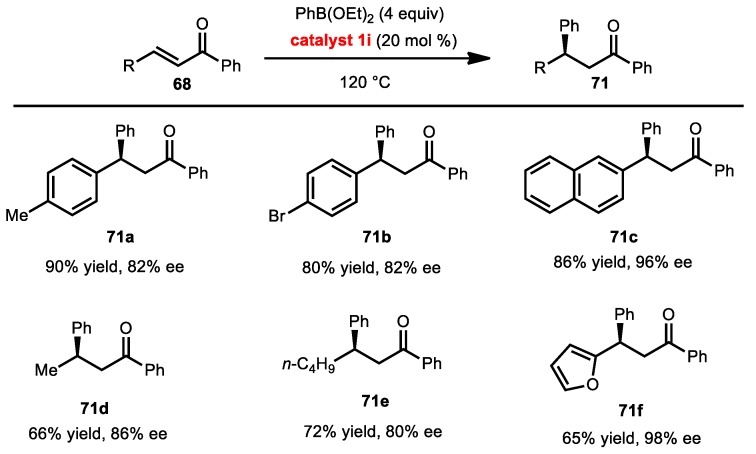
Conjugate addition of aryl boronic esters.

In 2011, the Chong group disclosed a conjugate addition of phenyl diethyl boronate using chlorinated BINOL catalyst [45]. In this study, the boronate ester was in excess amounts, to serve as solvent and provide excellent enantioselectivities and yields in most cases (Scheme 28). The reactions still proceeded well with most substrates. The greatest enantioselectivity was observed in the enone containing a 2-furyl substituent at the β-position to form **71f**. Additionally, the enone bearing a 1-naphthyl group also reacted smoothly and provided high enantioinduction in **71c**. Furthermore, the methyl and linear alkyl substituent at β-position were functional in these conjugate reactions with reasonable enantioselectivities.

**Scheme 29:**
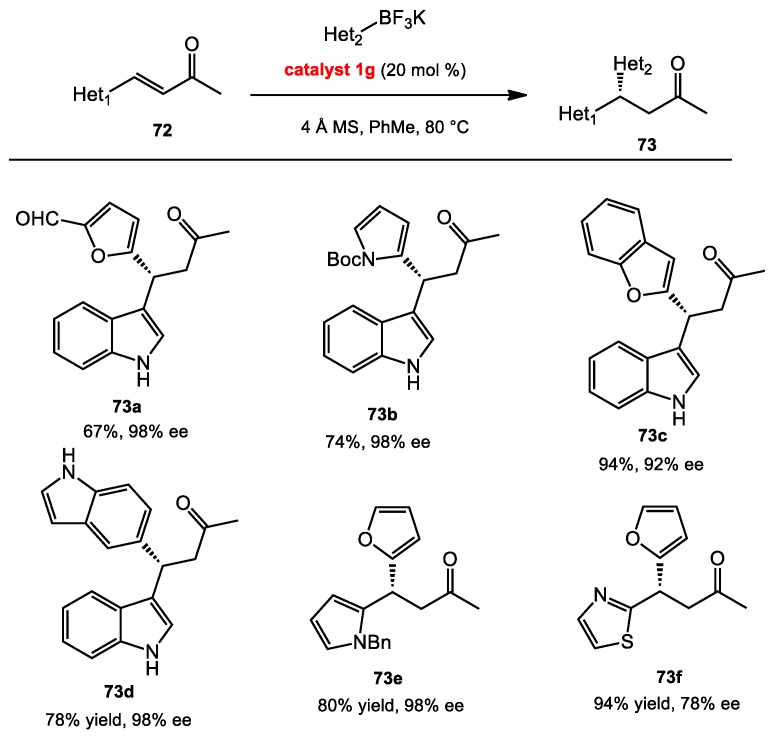
Conjugate addition of heteroaryl borate salts.

Later on, our group was interested in the use of heteroaryl and aryl nucleophiles in the conjugate addition (Scheme 29). We initially tested heteroarylboronic acids. However, low yields were obtained, and protodeboronation [46] was observed in noticeable amounts. To avoid this side reaction, organotrifluoroborate salts were used instead of boronic acids for their greater stability [47,48,49,50,51]. However, the insolubility of the organotrifluoroborates in nonpolar solvents was anticipated to be potentially problematic. Nevertheless, anhydrous nonpolar solvents turned out to be essential for the conjugate additions. Surprisingly then, the use of heteroaryltrifluoroborate salts provided products in excellent yields and enantioselectivities with heterocycle-appended electrophiles. Preliminary mechanistic investigations indicated that fluoride initially dissociated from the organotrifluoroborate salts, followed by the activation of the enones by the Lewis acidic BINOL organoborate complex, and then C–C bond formation. Moreover, molecular sieves were required to serve as a fluoride scavenger in the reaction [52,53].

Like the allylation, crotylation, and propargylation reactions, BINOL derivatives have served as excellent catalysts for many conjugate addition variants, including the use of *o*-quinone methides as especially electrophilic enones. We note, here, that tartrate derivatives also catalyze conjugate additions (see Section 3.3), but in comparing examples reported, to date, the use of BINOL derivatives is generally superior in both catalytic reactivity, to provide greater product yields, and in stereoselectivity, to provide products with greater enantiomeric excesses. However, the tartrate derivatives offer advantages in cost and facile synthetic access to highly variant derivatives that, when coupled with their significant reactivity and stereoselectivity, renders them a viable alternative to the axial chiral biaryl diols.

## 3. Tartaric Acid Derivatives

### 3.1 Asymmetric Addition of Alkenylborates to N-acyl Quinoliniums

**Scheme 30:**
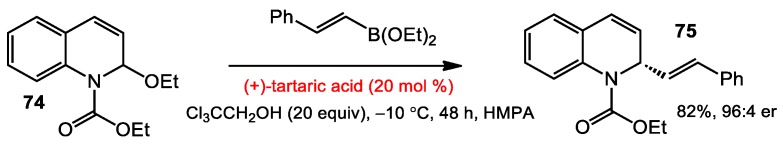
Enantioselective alkenylboronate addition to *N*-acyl quinoliniums.

In 2011, Schaus and co-workers reported the nucleophilic addition of alkenylboronates to 1-ethoxycarbonyl-1,2-dihydroquinolines (EEDQs) catalyzed by tartaric acid to afford enantioenriched dihydroquinoline derivatives (Scheme 30) [54]. The authors found that a mildly acidic additive, like Cl_3_CCH_2_OH, increased the reaction yield and enantioselectivity. Performing the reaction in HMPA at −10 °C improved the outcome, also. In general, the optimized conditions were suitable for many substituted EEDQ substrates, and vinylboronates in good yields (>70%) and enantioselectivities (>88:12% er).

**Scheme 31:**
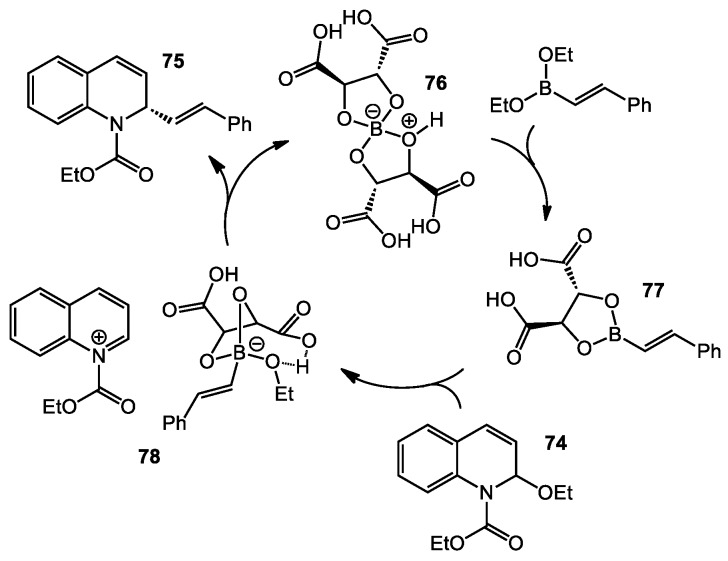
Proposed catalytic cycle for EEDQ addition.

Mechanistic studies were also undertaken to identify the role of tartaric acid (Scheme 31). The results revealed that dimeric tartaric acid adduct **76** was possibly formed. The boron “ate” complex **76** was independently synthesized and added to the reaction to serve as the catalyst. Under the optimized conditions, a similar outcome was observed. The borate **76** likely underwent ligand exchange to form boronate **77**. Next, the chiral alkenylboronate **77** reacted with the EEDQ to form the proposed intermediate **78**. The alkenyl group then migrated to the quinolinium enantioselectively to furnish the dihydroquinoline product.

### 3.2. Enantioselective Oxidative C–H Alkenylation and Arylation

**Scheme 32:**
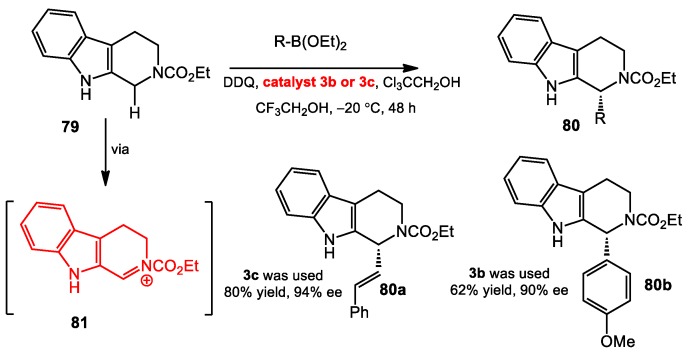
Enantioselective oxidative C–H alkenylation and arylation.

Liu and co-workers reported a similar transformation catalyzed by tartaric acid derivatives [55]. They described enantioselective C–H alkenylboration and arylboration reactions of tetrahydro-β-carbolines (carbolines) catalyzed by tartaric acid derivatives (Scheme 32). Inspired by the work of Schaus [39], they proposed that the cyclic iminium intermediates that could be selectively generated through benzylic C–H oxidation of the carboline precursors by DDQ could be intercepted in an enantioselective addition. The resulting *N*-acyliminium underwent a similar transformation to that reported by Schaus to afford diverse α-substituted tetrahydro-β-carbolines in good yields and high enantioselectivities. Notably, these additions to acyl quinoliniums performed significantly better with catalysts derived from tartaric acid. This may be due to the bimolecular nature of the C–C bond formation, as the allylations, crotylations, conjugate additions, and Petasis reactions that are most effectively catalyzed by BINOL and VANOL proceed intramolecularly, from an intermediate Lewis acid/Lewis base complex.

### 3.3. Asymmetric Conjugate Addition

**Scheme 33:**
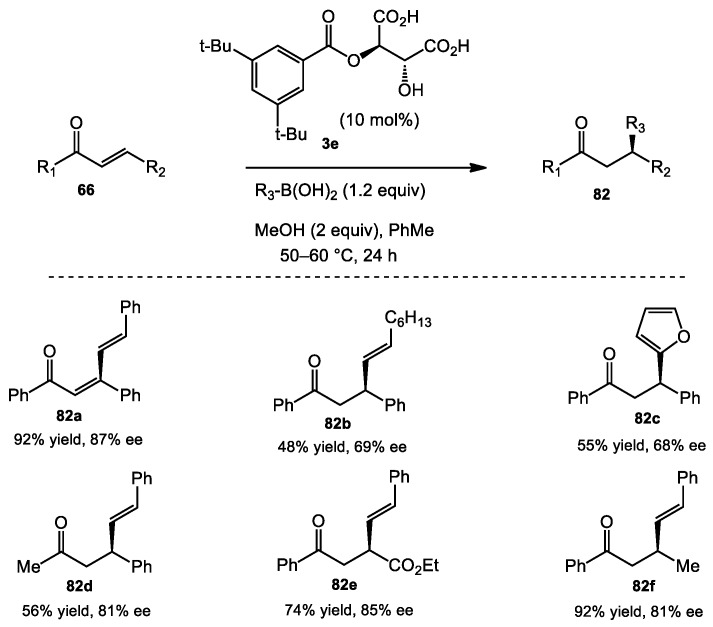
Asymmetric conjugate addition catalyzed by tartaric acid derivatives.

Tartaric acid derivatives were also employed to catalyze enantioselective conjugate additions. Sugiura developed a monoester tartrate catalyst **3e** for the conjugate addition of alkenyl boronic acids to enones (Scheme 33) [56,57]. During the course of optimization, they found that the addition of MeOH increased the rate of the reaction, presumably because of the formation of dimethyl boronate esters in BINOL-based catalysis. The optimized conditions allowed both aryl- and alkyl-substituted enones to be viable substrates (see **82a** and **82d**). Overall, yields and enantioselectivities of the reaction were moderate compared to those catalyzed by BINOL catalysts. However, a furan boronic acid nucleophile, an unprecedented example, was also reported (see **82c**).

**Scheme 34:**
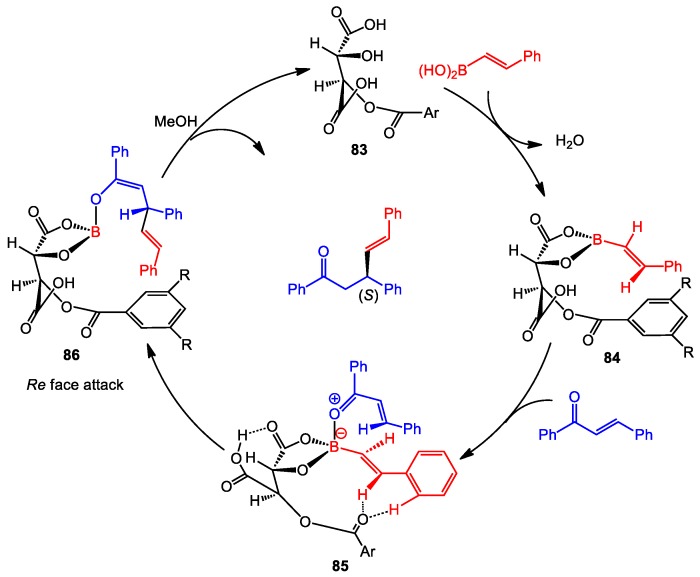
Proposed mechanism.

Mechanistic studies were also conducted, which indicated that the reaction mechanism is similar to that of the BINOL-catalyzed conjugate addition (Scheme 34) [58]. The boron “ate” complex **85** is presumably formed in the present of the catalyst. The internal hydrogen bond between two carboxylic acid groups appears to allow facial addition of the boronic acid nucleophile. The authors also suggested that non-classical hydrogen bonds between the benzoate carbonyl and the vinyl and aryl protons of the boronic acid, assist the transition state organization. After the addition of MeOH, the catalyst and the enone product are released and complete the catalytic cycle.

## 4. VANOL/VAPOL

BINOL **1**, a C_2_-symmetrical biaryl compound, has been widely applied in different reactions. Many derivatives have been synthesized in order to increase its reactivity and enhance the chiral pocket around the hydroxyl groups. While derivatives with substituents in the 3 and 3′ positions are common, variation in the scaffold that orients the hydroxyls is seen by the use of the biaryls VANOL **4** and VAPOL **5**. In 1993, Wulff’s group introduced VANOL and VAPOL as chiral ligands for a metal-catalyzed asymmetric Diels–Alder reaction [59]. During subsequent decades, many modified VANOL and VAPOL derivatives have been shown to be crucial in asymmetric reactions the aziridination of imines [60,61,62,63,64,65,66,67,68], the 2-aza-Cope rearrangement [69], the Ugi reaction [70], and other reactions [71]. Since this review is focused on diol organocatalysts, those reactions involving transition metals with VANOL or VAPOL ligands will not be covered here.

### 4.1. Petasis Reaction

**Scheme 35:**
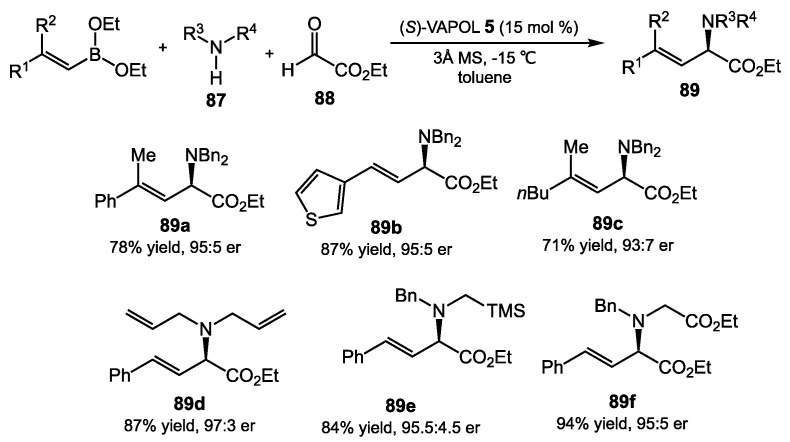
Asymmetric Petasis reaction catalyzed by VAPOL.

The Petasis reaction is an extremely useful transformation, in which an aryl, alkenyl, or alkynyl organoboronate is condensed with amines and aldehydes to give a branched alkyl amine [72,73,74]. The Schaus group disclosed an asymmetric Petasis reaction catalyzed by (*S*)-VAPOL **5** to offer α-amino esters **89** in good yield and high enantiomeric excess [75]. During the exploration of catalyst structures, they tested a series of BINOL derivatives with a variety of additional functional groups at the 3 and 3′ positions; however, none of them provided a better enantioselectivity than (*S*)-VAPOL **5**. The optimal conditions could be utilized for many boronates, including those containing alkenyl groups, heteroaromatic-substituted alkenyl groups, alkyl groups, a variety of secondary amines **87**, and ethyl glyoxylate **88** (Scheme 35). We note that this reaction is substantially similar to the BINOL-catalyzed addition of aryl and allyl boronate esters to acyl imines in Section 2.3. In general, the axially chiral aromatic diols **1**, **2**, **4**, and **5** are catalytically competent in this reaction, and the best catalyst for both a high yield of the product and the greatest stereoselectivity is typically determined empirically.

### 4.2. Aziridination

**Scheme 36:**
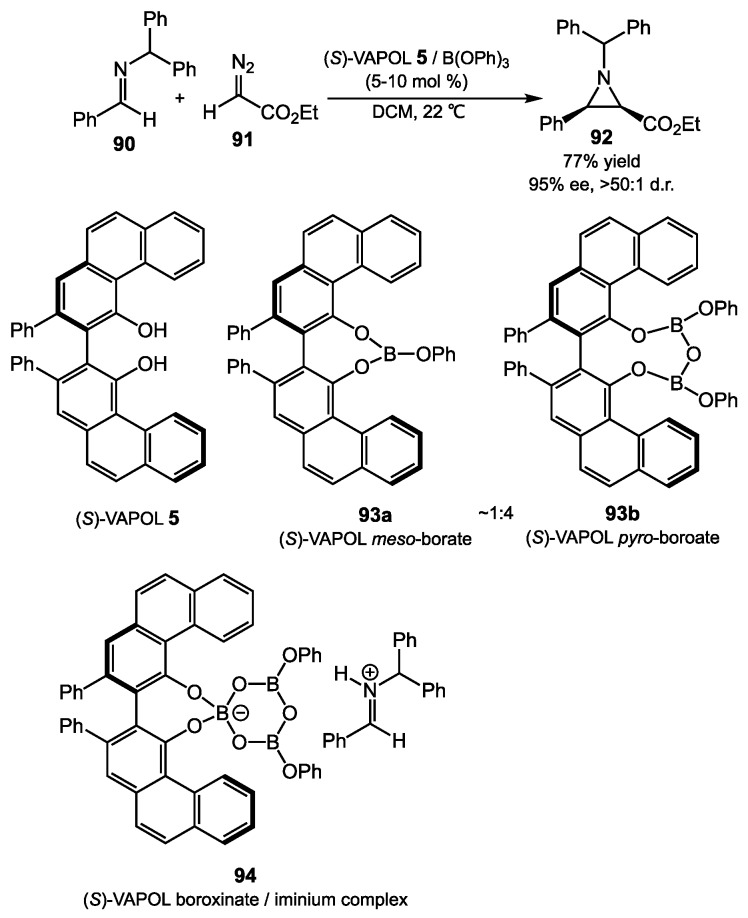
Asymmetric aziridination reaction with VAPOL iminium boroxinate.

The Wulff group demonstrated that the mixture of (*S*)-VAPOL **5** and B(OPh)_3_ gives (*S*)-VAPOL *meso*-borate and *pyro*-borate complexes **93a** and **93b** in approximately a 1:4 ratio. The mixture could be utilized as a precatalyst in the synthesis of aziridines **92** from the combination of imines **90** and diazoacetates **91** (Scheme 36). A comprehensive study on asymmetric aziridination reaction with BINOL boroxinates, including the reaction scope, mechanism, and synthetic applications, was also reported by the Wulff group recently [71]. However, the BINOL complexes gave lower yields and stereoselectivities than VANOL and VAPOL derivatives. In general, the Lewis acidic active catalyst was pre-formed by mixing the diol with B(OPh)_3_ before the reactants are added. While the reaction does not directly use any organoboronates as a reagent, as is seen in the above examples, spectroscopic and crystallographic studies indicated that the active intermediate was the ion pair complex **94**, which could be generated from treatment of the mixture of borate **93a** and **93b** with imine **90** [63,76]. The proximity of the ions in the nonpolar solvent presumably allows VAPOL adduct **94** to control the facial selectivity of both the imine and diazoester. This method could provide highly diastereoselective and enantioselective aziridines, and has been applied in the synthesis of the precursors of florfenicol and β^3^-homo-amino acid [60,77].

### 4.3. Protonation of Silyl Enol Ethers

**Scheme 37:**
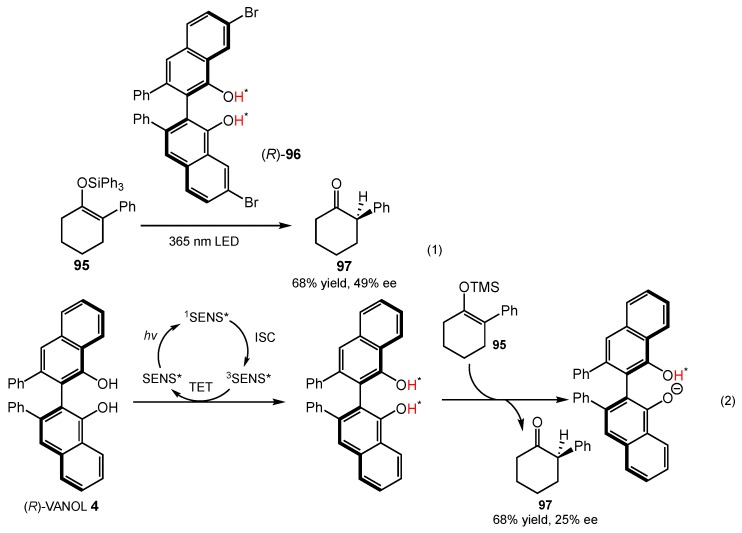
Enantioselective protonation of silyl enol ethers.

In 2016, Hanson and coworkers showed that (*R*)-3,3′-Br_2_-VANOL **96** could act as an enantiopure excited state proton transfer (ESPT) dye under 365 nm irradiation, which could be used for the asymmetric protonation of a range of silyl enol ethers **95** (Scheme 37, eq 1) [78]. By using (*R*)-3,3’-Br_2_-VANOL **96**, up to 49% ee and a 68% yield of products was observed at room temperature; however, no ee was obtained when using BINOL **1a**, BINOL derivative **1b**, or VANOL **4**. According to their previous work [79], the same transformation could also be promoted under visible light (445 nm) by the addition of appropriate triplet sensitizer (SENS), such as bis(4,6-difluorophenyl-pyridine) (picolinate)-iridium (III) (Scheme 37, eq 2). Irradiation of a mixture of a silyl enol ether **95**, (*R*)-VANOL **4**, and bis(4,6-difluorophenyl-pyridine) (picolinate)-iridium (III) in toluene with 445 nm light produced ketone **97** in 68% yield with 25% ee. Based on the results of circular dichroism, HPLC, and UV–vis spectroscopy, Hanson’s group reasoned that the relatively low ee of the protonated product could be attributed to the racemization/decomposition of the ESPT dye (*R*)-VANOL **4** in the excited state.

## 5. Silanediol

The silanediol functional group, a silicon with geminal diol attached, has been capturing chemists’ attention because it is an excellent motif to present hydrogen bond donors [80]. Early work from Kondo and coworkers reported that recognition of acetate, chloride, and bromide was possible for silanediol in 2006 [81]. Mattson’s group showed that achiral silanediols could activate nitroalkenes for nucleophilic attack [82]. Franz and coworkers demonstrated the catalytic activation of carbonyl compounds by silanediols in 2011 [83]. In this section, we will focus on enantioselective transformations of organic compounds by silanediol catalysts.

### 5.1. N-acyl Mannich Reaction

**Scheme 38:**
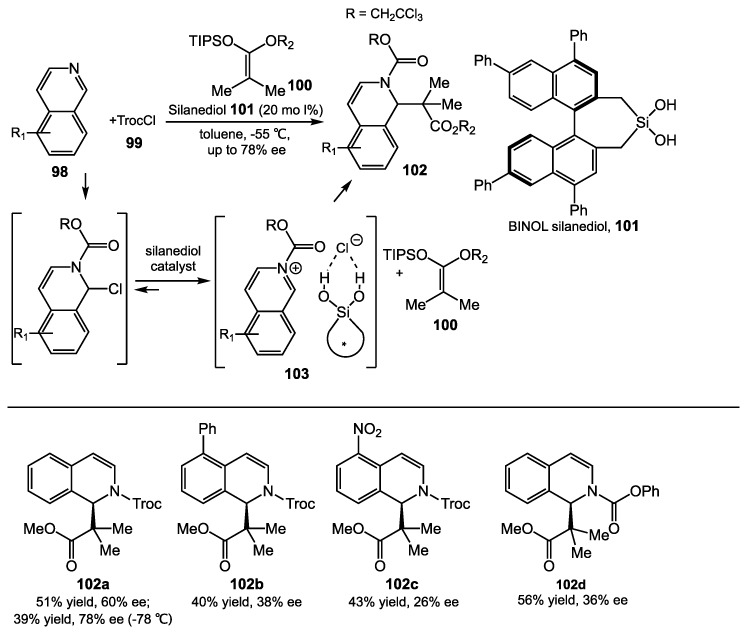
Enantioselective acyl Mannich reaction with silanediol catalysis.

Mattson and coworkers first introduced enantioselective silanediol halide-binding catalysis in an acyl Mannich reaction in 2013 [84]. It was proposed that the chiral ion pair **105** was generated in situ from *N*-acylisoquinoline **98** with TroCl by silanediol halide binding, which forms a coordinated counterion that controls the enantioselective bond formation. The addition of the silyl ketene acetal **106** to the intermediate **105** can afford product **107** in nearly 80% ee. With a library of cyclic and acyclic BINOL- and VANOL-derived silanediols, it was suggested that optimizing results can occur with variation of the backbone of the silanediol (Scheme 38) [85,86].

### 5.2. Chromenone Functionalization

**Scheme 39:**
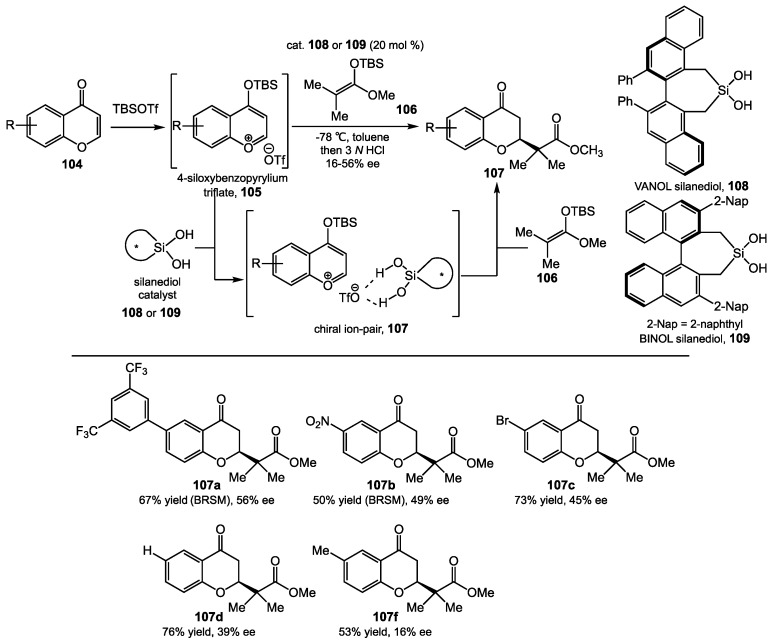
Alkylation reactions of various chromenones.

In 2016, Mattson and Kondo reported promising levels of enantioselectivity in the silanediol-catalyzed addition of silyl ketene acetals **106** to benzopyrylium triflate intermediates like **105** [87]. The key to the success of this methodology was the capture of 4-siloxybenzopyrylium triflates **105** with a chiral silanediol **108** or **109**, that resulted in the in situ generation of a chiral ion pair **107**. Mattson and Kondo proposed that this transition state would lead to the facially biased addition of nucleophiles to the benzopyrylium ion. In order to find a suitable chiral environment to stabilize the transition state, they tested a serious of arene-rich silanediols, such as cyclic BINOL-based silanediols and both cyclic and acyclic VAPOL-based silanediols. Fortunately, a 3,3′-bis-2-naphthyl BINOL-based silanediol **109** with a bulky group (i.e., 2-naphthyl) provided both high yield and good stereoselectivity for the functionalization of chromenones. The VAPOL-based silanediol **108** could also serve as a catalyst for this reaction. They reasoned that increasing the bulkiness surrounding the silanediol could prevent undesired silylation, and reaction results were indeed greatly affected by the structure of the chromenone. Additionally, chromenones possessing electron-withdrawing groups gave higher yield and higher levels of enantioselectivity than chromenones with electron-donating groups. The chromenones with a 3,5-bistrifluoromethylphenyl or nitro in the 6-position (Scheme 39, **107a** and **107b**) provided the highest enantioselectivity (56% ee and 49% ee, respectively). On the other hand, a hydrogen or methyl group in the 6-position only gave 39% ee and 16% ee, respectively (Scheme 39, **107d** and **107f**). This reactivity is promising for the synthesis of bioactive chromanones and tetrahydroxanthones. Silanediol derivatives of the tartrate platform were not reported, so currently, the axially chiral biaryl platform is the most functional option for the development of silanediol catalysts, with the BINOL-based catalysts performing the best.

## 6. TADDOL Derivatives

### 6.1. Hydrocyanation of Aldimides

**Scheme 40:**
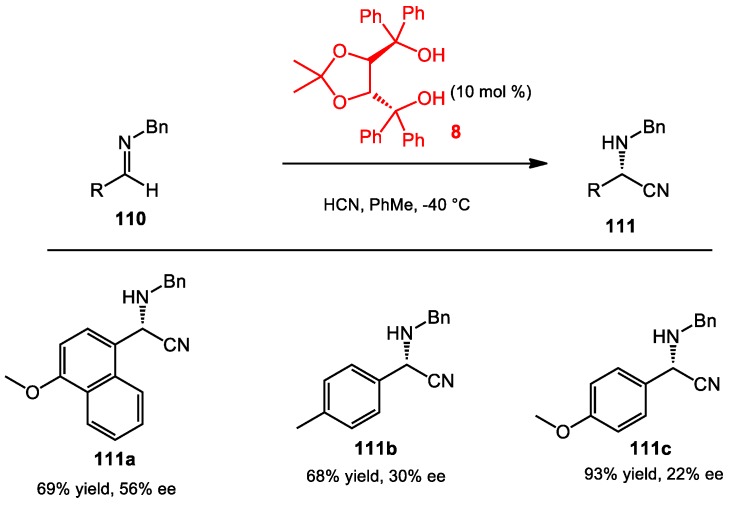
Hydrocyanation catalyzed by TADDOL.

Rueping studied the addition of hydrogen cyanide to imines, also known as the Strecker reaction, in 2006 [88]. Varieties of BINOL phosphates were shown to provide high yield and enantioselectivity in this transformation. Later on, Rueping also investigated the Strecker reaction with ketoimine substrates, leading to the synthesis of quaternary amino acids [89]. Initially, various BINOL phosphates were screened. These studies showed that a BINOL with 9-phenanthryl substitution at the 3,3′-positions provided high yields and enantioselectivities with the ketoimine substrates. Furthermore, TADDOL **8** also was found to be a promising Brønsted acid to catalyze the Strecker reaction with imine substrates (Scheme 40). However, enantioselectivities were moderate with the use of this catalyst.

### 6.2. Diels–Alder Reaction

**Scheme 41:**
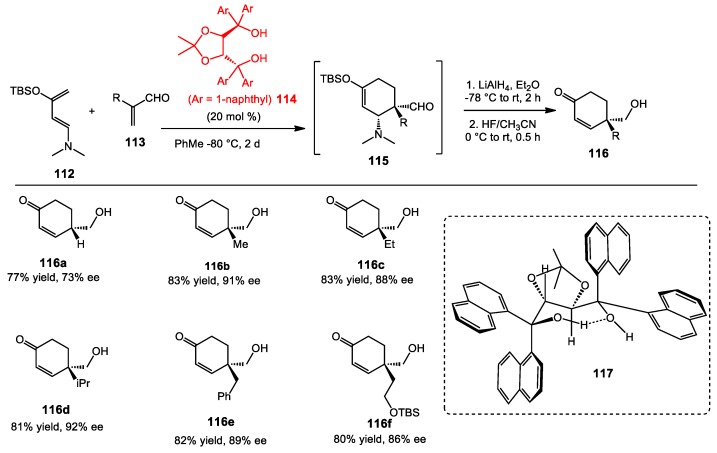
TADDOL-catalyzed enantioselective Diels–Alder reaction.

Although hydrogen bond-activated reactions have been studied broadly, examples of stereoselective transformations are relatively sparse, especially for the Diels–Alder reaction [90,91,92,93,94]. In 1990, the Kelly group reported the Diels–Alder reaction of cyclopentadiene with several acroleins and acrylates, using an achiral biphenylenediol as the catalyst [95]. The diol accelerated the reaction rates through the complexation of the carbonyl group and the diol through two hydrogen bonds. In 2000, the Göbel group disclosed the enantioselective Diels–Alder reaction using hydrogen-bonding coordination of a dienophile and a chiral amidinium ion [96]. Later on, the Rawal group was interested in H-bonding-mediated reactions, and they reported a diol-catalyzed enantioselective Diels–Alder reaction (Scheme 41) [97]. TADDOL **114** was used as a Brønsted acid catalyst, which acted as the H-bonding activator of the dienophiles. Furthermore, the aryl substituents of the TADDOL played an important role in the intramolecular H-bonding between the two hydroxyl groups, resulting in the formation of the seven-membered ring in the 3D representation **117**. The spatial orientation of TADDOL dramatically affected the enantioinduction by forming strong complexes with Lewis basic atoms. A TADDOL with a 1-naphthyl substituent did not have free rotation around the carbon–naphthyl bonds, leading the authors to expect a greater ability to form an intermolecular H-bond with the carbonyl groups of the dienophile. Varieties of dienophiles were tested and, in most cases, gave high yields and enantioselectivities. Additionally, preliminary studies showed that the reactions proceeded favorably with α-substituted acroleins. To explain the absolute configuration of the product, the free hydroxyl group of TADDOL was proposed to form H-bonding with carbonyl groups, leading to nucleophile attack at the *si*-face of the aldehyde. The yields and stereoselectivities for this reaction were later improved with the development of the BAMOL catalysts (Section 7).

### 6.3. Vinylogous Mukaiyama Aldol Reaction

**Scheme 42:**
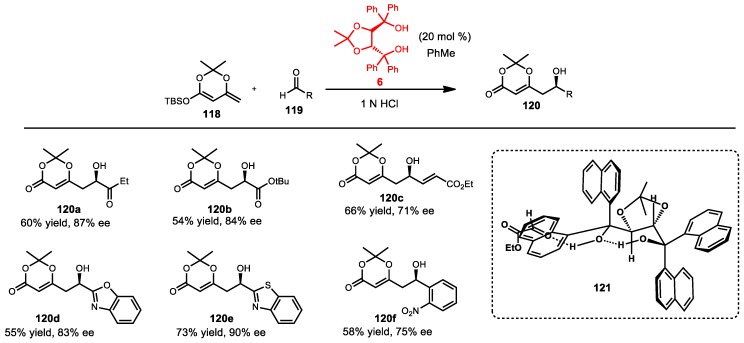
TADDOL-catalyzed vinylogous Mukaiyama aldol reaction.

Enantioselective vinylogous Mukaiyama aldol reactions are useful methods for organic syntheses. This reaction has been developed and implemented widely during the past decade. Several methods were reported using Lewis acidic metals, such as boron [98], titanium [99,100,101], copper [102,103,104], and chromium [105]. Metal-free vinylogous Mukaiyama aldol reactions have been rarely developed and reported. Rawal first introduced a hydrogen bond-catalyzed enantioselective vinylogous Mukaiyama aldol in 2005 [106]. Chiral diol catalysts were screened for the enantioselective vinylogous Mukaiyama aldol reaction. TADDOL **6** provided the greatest yield and enantioselectivity. Various aldehydes were tested in this transformation. Most aromatic aldehydes exhibited moderate yields and enantioselectivity. However, aliphatic aldehydes were less reactive, and the acceptable yields and levels of enantioselectivity were observed. The proposed transition state indicated that the TADDOL controlled the hydrogen bond with the aldehyde oxygen through π–π* donor–acceptor interactions between the equatorial 1-naphthyl ring and the aldehyde carbonyl **121** (Scheme 42).

### 6.4. Nitroso Aldol Reaction

**Scheme 43:**
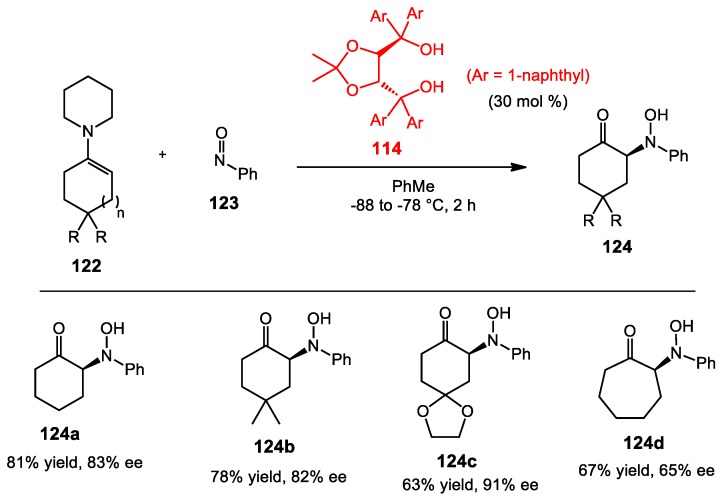
Asymmetric nitroso aldol reaction.

The nitroso aldol reaction is a useful approach to synthesize α-amino carbonyl products. Lewis first reported the nitroso aldol reaction in 1972 [107]. The aldol adduct was obtained in acceptable yield. Later on, Yamamoto introduced a modification to provide greater yields using the pyrrolidine enamine of cyclohexanone (Scheme 43) [108]. Furthermore, they found that Brønsted acid played an essential role to expedite the reaction. Various enamines and TADDOLs were used to study the enantioselective nitroso aldol reaction. TADDOL catalysis provided excellent yields and enantioinduction in most cases.

### 6.5. Mukaiyama Aldol Reaction

**Scheme 44:**
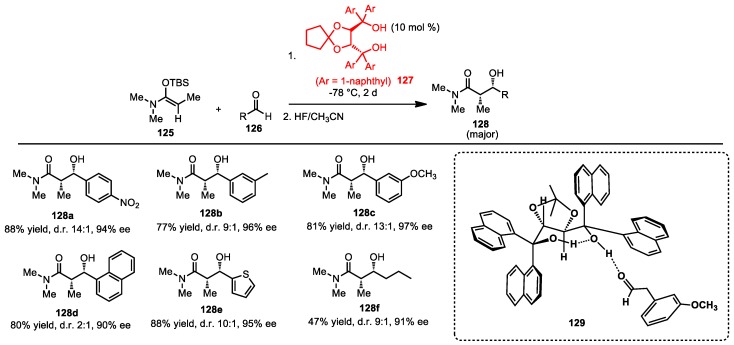
Enantioselective Mukaiyama aldol reaction.

After the successful use of H-bond catalysis in enantioselective vinylogous Mukaiyama aldol reactions in 2005, the Rawal group was interested in the diasteroselective Mukaiyama aldol reaction using H-bond activation. Many well-known methods using chiral metal-based Lewis acids have been reported [109,110,111]. Metal-free-based Mukaiyama aldol reactions have been rarely reported, but in 2006, the Rawal group reported such a reaction (Scheme 44) [112]. Previously, the Rawal group found that TADDOL **127** showed promising activity in vinylogous Mukaiyama aldol. Here, treatment of *O*-silyl-*N*,*O*-acetal **125** with benzaldehyde in the presence of TADDOL **127** generated the aldol adduct in high yield and stereoselectivity. Decreasing the reaction temperature significantly improved the diastereo- and enantioselectivities. The para—**128a** and meta—**128b**, **128c** substituents on the aldehyde phenyl ring reacted well under the H-bond catalysis of the TADDOL. Aldehyde substrates with naphthalene **128d** or thiophene **128e** also provided products in excellent yields and stereoselectivities. Interestingly, preliminary results showed that treatment of aliphatic aldehydes with TADDOL gave the aldol adduct **128f** in high enantioselectivity, but a moderate yield was observed.

### 6.6. Mukaiyama Aldol Reaction of Ketoesters

**Scheme 45:**
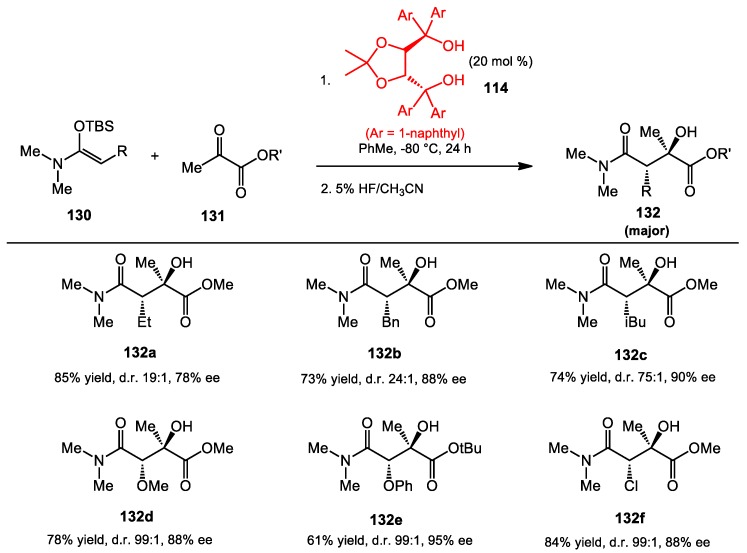
Asymmetric Mukaiyama aldol reaction of ketoesters.

The stereoselective Mukaiyama aldol reactions with ketone substrates have been reported using metal-based complexes by the Evans group [113]. In 2010, the Rawal group reported the further development of metal-free diastero- and enantioselective Mukaiyama aldol reactions with ketone substrates (Scheme 45) [114]. The *N*,*O*-ketene acetal was initially treated with alkyl-substituted pyruvates to generate aldol adducts in high yields in the presence of naphthyl-TADDOL catalyst **114**. The studies showed that alkyl-substitution on the ketene acetals did not limit reactivity. Larger alkyl groups **132c** provided products with greater stereoselectivity. Likewise, the reactivity of the pyruvate was improved with the larger alkyl-substituents **132e**. Various hetero-atom *α*-substituents **132e** and **132d** on the ketene acetal also were accommodated by the TADDOL-catalyzed Mukaiyama aldol reaction. High reactivities and stereoselectivities were obtained in most cases. With the exception of the hetero-Diels–Alder reaction, the TADDOL-catalyzed reactions presented here have not been examined with other diol catalysts and, thus, there may be catalyst alternatives that work as well or better.

## 7. BAMOL

**Scheme 46:**
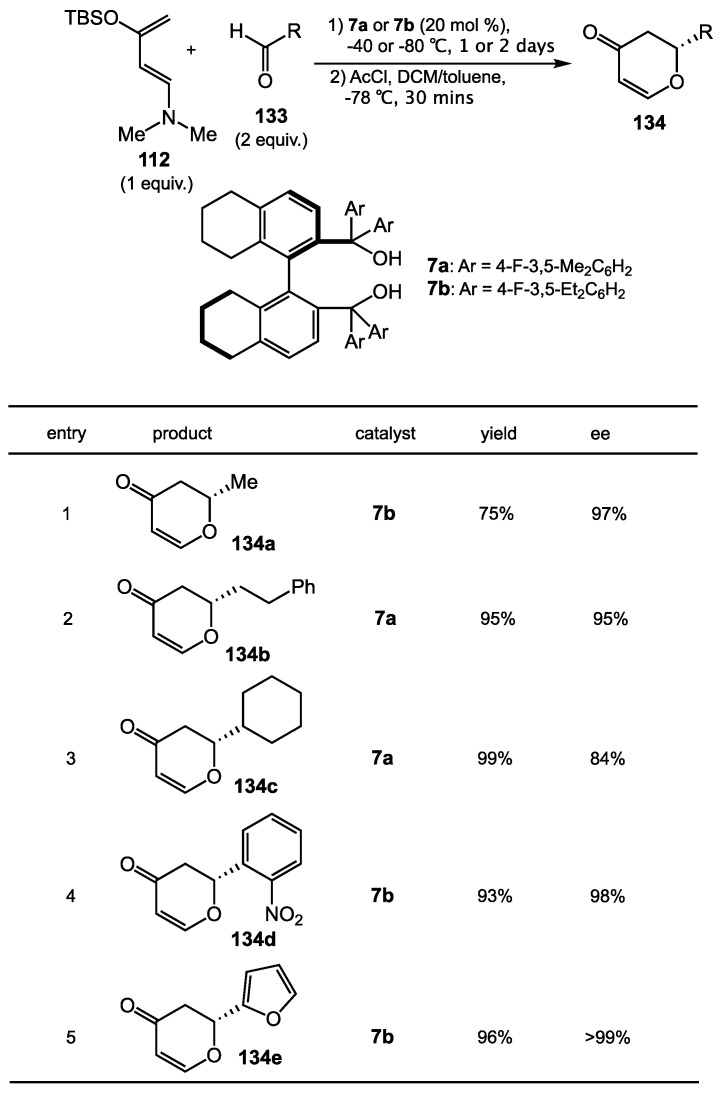
Asymmetric Hetero-Diels–Alder reaction Catalyzed by BAMOL.

The hetero-Diels–Alder (HDA) reaction could be catalyzed by TADDOL, a chiral diol, through hydrogen bonding (Section 6.2). Inspired by the structure of TADDOL, the Rawal and Yamamoto groups reported in 2005 a new hydrogen bonding catalyst, 1,1′-biaryl-2,2′-dimethanol (BAMOL, **7**) that produced products in higher yields than TADDOL for a highly enantioselective HDA reaction (Scheme 46) [115]. This scaffold shares the same bis(diarylhydroxymethyl) functionality with TADDOLs with the bisaryl axial chirality seen in BINOL and VAPOL, and the steric and electronic properties can be modified via a three-step synthesis. BAMOLs with 4-fluoro-3,5-dimethylphenyl and 4-fluoro-3,5-diethylphenyl groups (**7a** and **7b**) gave the best yields and enantioselectitivities during a survey of BAMOL catalysts. With the BAMOLs and conditions they developed, 1-amino-3-siloxybutadiene **133** effectively reacted with wide range of aldehydes, including straight-chain aliphatic, heteraryl, and electron-poor derivatives to give HDA products **134** in good yields and high enantioselectivities.

## 8. Recent Developed Chiral Diols

### 8.1. Chiral Ferrocenyl Diols

**Scheme 47:**
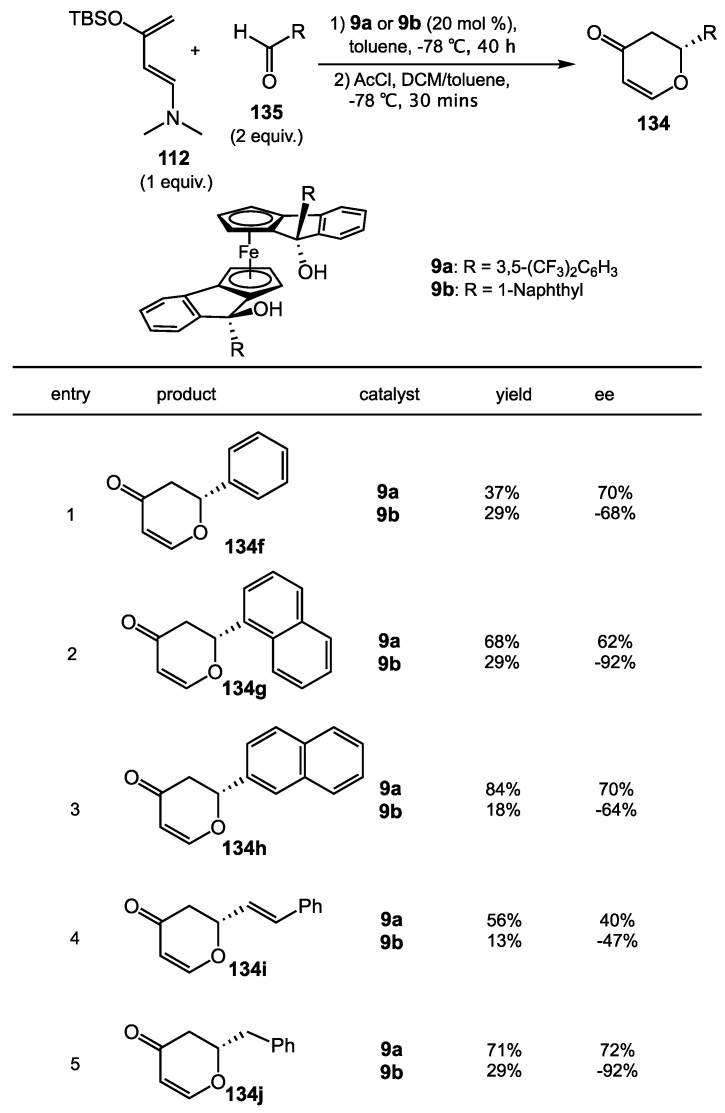
Asymmetric hetero-Diels–Alder reaction catalyzed by chiral ferrocenyl diols.

In the field of enantioselective organocatalysis, it is important to have an easy modular and tunable chiral motif. In 2016, the Guiry group presented a short synthesis of ferrocenyl-based diols **9a** and **9b**, where many derivatives could be obtained from the same synthetic strategy [116]. The family of ferrocenyl diols possesses many advantages: planar chirality on both ferrocenyl cyclopentadienyl rings, central and axial chirality around the iron center, and rotational flexibility in the ferrocenyl backbone. The X-ray crystal structure of ferrocenyl-diols revealed that they have similar type of hydrogen bonding as TADDOL in the solid state. With the concept of reaction activation via hydrogen bonds in mind, the Guiro group utilized ferrocenyl-diols **9a** and **9b** in the hetero-Diels–Alder reaction between 1-amino-3-siloxybutadiene **112** and aldehydes **135** (Scheme 47). Different aldehydes were examined, including phenyl, naphthyl, vinyl, and alkyl. They provided cycloadducts **134f** and **134j** in up to 84% yield with good ee values (70-92%), though the results were generally significantly worse than those obtained from the BAMOL catalysts. It is interesting that the opposite enantiomer of product **134** could be obtained by using catalyst **9a** or **9b** under the same reaction conditions. This observation could be accounted for the naphthyl group on the catalyst **9b** has a greater steric interaction to the R group of the aldehyde **135** then the 3,5-bis(trifluoromethyl) benzyl group on the catalyst **9a**. Detailed possible transition states were discussed in their report. In all, this is a novel chiral diol concept that merits additional exploration.

### 8.2. HAROLs

**Scheme 48:**
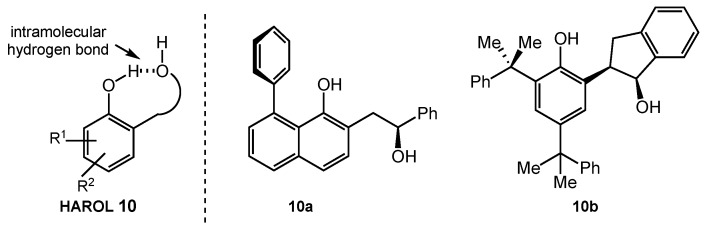
Structures of hydroxyarylalcohols (HAROLs).

**Scheme 49:**
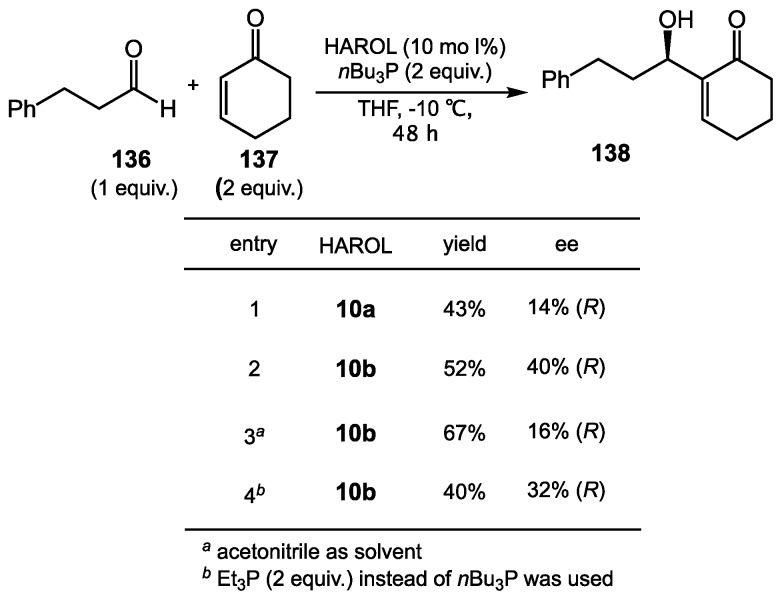
Asymmetric Morita–Baylis–Hillman reaction catalyzed by HAROLs.

The Ertirk group reported efficient syntheses of new chiral 1,4-diols possessing one phenolic and one aliphatic hydroxyl group (i.e., 2-(2-hydroxyaryl)alcohols, HAROLs **10**) in 2018 (Scheme 48) [117]. Within the HAROL-type 1,4-diol carrying two hydroxyl groups, intermolecular hydrogen bonds may form with electrophiles such as aldehydes and ketones. As proof of this concept, the Ertirk group demonstrated an asymmetric Morita–Baylis–Hillman reaction between aldehyde **136** and cyclohexenone **137** in the presence of 10 mol% of HAROLs (Scheme 49). Among the library of HAROLs they examined, catalysts **10b** worked the best to afford the product **138** in 52% yield and with 40% ee (entry 2). They also pointed out that changing the solvent from THF to acetonitrile improved the reactivity of catalyst **10b** to give higher yield, but the enantioselectivity decreased dramatically (16%) (entry 3). Their catalytic system became ineffective if other nucleophilic promoters, such as DABCO, DBU, DMAP, and other phosphines, were present. While the catalyst design was innovative and was used to explore several transformations, it did not approach the reactivity or stereoselectivity in the Morita–Bayless–Hillman reaction that Schaus’s group achieved with BINOL derivatives [118].

## 9. Conclusions

Organocatalysis offers an orthogonal approach to metal-based catalysis, and has advantages in avoiding toxic elements, avoiding catalyst poisoning, delivering high functional group tolerance, and even allowing access to novel transformations. Chemists are able to leverage modern synthetic knowledge to access reactive chiral diols that can serve as catalysts for many useful transformations. This review introduces many recent developments in this area with the goal of inspiring even more innovation in chiral diol catalysis.

Generally, the biaryl diols **1**, **4**, and **5** have performed the best for the most examples, though tartrate derivatives should not be overlooked as they often perform better. As many reports of reaction development focus on a single scaffold class, it is often difficult to directly compare catalyst performance. In those cases where a direct comparison was possible, it has been discussed in the pertinent sections.

It is also important to observe that substituents adjacent to the hydroxyls of the diols have a significant impact on reactivity and stereoselectivity. BINOL derivatives, where substituents at the 3 and 3′ positions are straightforward to synthesize, thus offer a powerful scaffold platform for a catalyst library that exhibits great steric and electronic diversity to probe for new reactivity. The tartaric acid foundation allows for modification of one of the hydroxyls, for transformation of the carboxylic acid to various esters and amides, and for access to TADDOL derivatives. Through these transformations, diversity in the steric and electronic environment of the key hydroxyls may be controlled.
